# Synthesis and Biological Evaluation of Novel 2‐(Piperidin‐4‐yl)‐1,2,3,4‐tetrahydroisoquinoline and 2‐(Piperidin‐4‐yl)decahydroisoquinoline Antimycotics

**DOI:** 10.1002/ardp.70128

**Published:** 2025-10-16

**Authors:** Marie‐Christin Hain, Monika Klimt, Franz Bracher, Ulrike Binder, Jürgen Krauß

**Affiliations:** ^1^ Department of Pharmacy, Center of Drug Research Ludwig‐Maximilians University Munich Munich Germany; ^2^ Institute of Hygiene and Medical Microbiology, Department of Hygiene, Microbiology and Virology Medical University Innsbruck Innsbruck Austria

**Keywords:** 4‐aminopiperidine, antifungals, *Candida* spp., reductive amination, yeasts

## Abstract

Piperidine antimycotics like fenpropidin are well established in agrochemistry. On the other hand, numerous isoquinoline derivatives show remarkable antimycotic effects. Here we present a series of 13 hybrid molecules of both lead structures, which were prepared using reductive amination as key reaction step. Pre‐screening against *Yarrowia lipolytica* resulted in a batch of promising candidates whose antifungal efficacy was further evaluated against clinically relevant species. In these assays, complete growth inhibition was seen for five or six compounds against *C. albicans* or *C. krusei*, respectively, and in two against *C. glabrata*, whereas no antifungal activity was observed against mold isolates, with the exception of **6i**, which led to complete growth inhibition of aspergilli, and two compounds (**6k** and **6 l**) that were able to inhibit *Rhizopus arrhizus*.

## Graphical abstract

A series of 13 hybrid molecules of both lead structures were prepared using reductive amination as key reaction step. The antimycotic potency of the resulting compounds was evaluated against yeast and mold species. Two compounds showed remarkable potency against several yeasts, and even against *Candida krusei*.

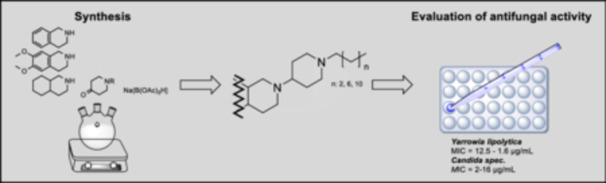

## 1 Introduction

Invasive fungal infections are an increasing problem in modern medicine especially for immunocompromised and hospitalised patients. Patients with AIDS, organ‐grafted patients, patients under cytostatic regimes or after stem cell therapy have a high risk of systemic fungal infections, and often a prophylactic therapy with antimycotics is necessary. Among the authorized medical substances, there are currently only four major groups of antimycotics available against systemic mycosis. These are the azoles (mainly fluconazole, voriconazole, posaconazole, and isavuconazole), the polyenes with amphotericine B in various modern formulations, the echinocandines (caspofungin, anidulafungin, micafungin and rezafungin) and the antimetabolite flucytosine [1, 2, 3]. Only a few substances with new mechanism of action like olorofim (F901318) [4] are in development. On the other hand, an increasing resistance against these antimycotics makes therapy increasingly difficult. Azole‐resistant strains of *Candida* or *Aspergillus* have been isolated from patients with higher frequency, as well as echinocandin‐resistant *Candida* strains. Furthermore, also among fungi causing superficial infections, antifungal resistance is increasingly observed, such as in terbinafine‐resistant dermatophytes [5, 6, 7, 8]. These findings are worrying and highlight the need for a broader arsenal of antifungal agents that could be developed for clinical use.

Piperidine antimycotics like fenpropidin (**A**) are broadly used in agriculture. They are potent ergosterol biosynthesis inhibitors and their mechanism of action is similar to the morpholine class (like amorolfine (**B**) or fenpropimorph) by inhibition of two enzymes of ergosterol (**C**) biosynthesis: the Δ14‐reductase and C7/8‐isomerase [9, 10]. Interaction with these enzymes leads to depletion of ergosterol and accumulation of toxic ergosterol precursors, resulting in altered membrane permeability and, ultimately, cell death. MIC values against *Candida* species are reported for fenpropidin with about 0.25–1 µg/mL and fenpropimorph with 0.5–2 µg/mL. By now no piperidine antimycotics are used in human therapy and the related morpholine amorolfine is only used in topic formulations [11].

**Figure 1:**
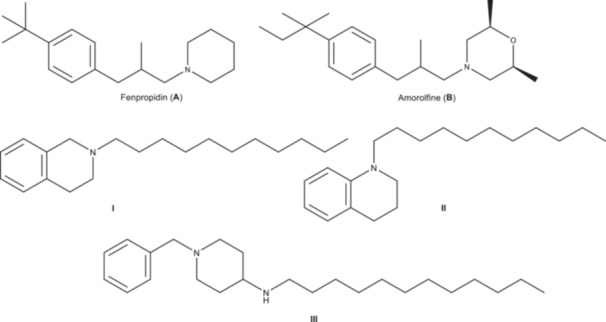
Structures of established antifungals and our earlier research compounds. Piperidines/morpholines: fenpropidin (A), amorolfine (B), antifungal compounds from our previous work: **I** [13], **II** [14], and **III** [15].

Furthermore, numerous tetrahydroisoquinoline and decahydroisoquinoline as well as tetrahydroquinoline and decahydroquinoline derivatives show remarkable antimycotic effects against *Candida* species [12]. In earlier work we found that simple *N*‐alkyl tetrahydroisoquinolines (**I**) and *N*‐alkyl‐decahydroisoquinolines as well as their tetrahydroquinoline (**II**) and decahydroquinoline analogues (Figure 1) show remarkable antimycotic potency in correspondence to their alkyl chain length with a maximum activity with an unbranched C_11_ chain length with MIC values of 5 µg/mL against *Candida glabrata* [13, 14, 16, 17]. Furthermore, we found recently that 4‐aminopiperidine derivatives (**III**) show noteworthy antimycotic activity against *Candida* and *Aspergillus* species with MIC values of about 1‐4 µg/mL [15].

**Figure 2:**
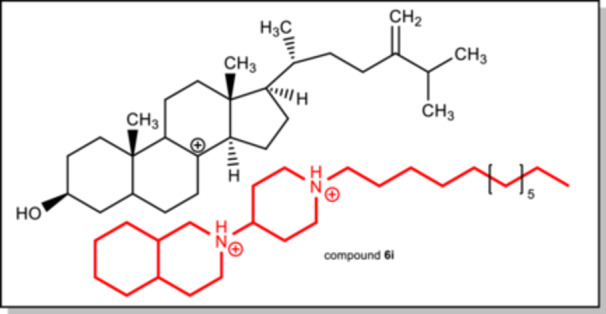
Structures of a precursor of episterol (HEI) and protonated active compound **6i**.

In continuation of our research on novel antimycotics, we present here the synthesis and antimycotic evaluation of a series of *N*‐alkylpiperidin‐4‐yl substituted tetrahydro‐ and perhydroisoquinolines. These compounds are hybrid molecules of our previously identified antifungal 4‐aminopiperidines and *N*‐alkyl tetra‐ and perhydroisoquinolines and might mimic the ergosterol skeleton or more exact high‐energy intermediates (HEI) of ergosterol biosynthesis (Figure 2) as shown for amorolfine [11]. Only the long‐chain 2‐(*N*‐alkylpiperidin‐4‐yl)‐1,2,3,4‐tetrahydroisoquinolines and 2‐(*N*‐alkylpiperidin‐4‐yl)decahydroisoquinolines show interesting antimycotic activity against pathogenic and non‐pathogenic yeasts. Their activity is strongly depending of the length of the alkyl side chain and a protonatable amine function in the piperidine ring. Their corresponding amides showed no remarkable activity as well as their homologues with a four carbon atoms containing side chain.

The compounds obtained by this strategy were evaluated for their antimycotic potency against several yeasts and molds, including clinically relevant species.

## 2 Results and Discussion

### 2.1 Chemistry

**Scheme 1:**
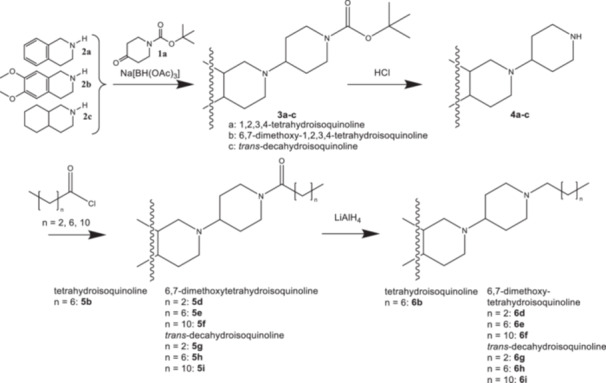
Synthesis of the compound series **3**, **4**, **5**, and **6**.

Commercially available *tert*‐butyl 4‐oxopiperidine‐1‐carboxylate (**1a**) was reacted in a reductive amination with the secondary amines 1,2,3,4‐tetrahydroisoquinoline (**2a**), 6,7‐dimethoxy‐1,2,3,4‐tetrahydroisoquinoline (**2b**) or *trans*‐decahydroisoquinoline (**2c**) and sodium triacetoxyborohydride [15, 18, 19] to give the corresponding Boc‐protected diamines **3a‐c**. After cleavage of the Boc protective group with hydrogen chloride in diethyl ether the free secondary amines **4a‐c** were converted with aliphatic linear (C_4_, C_8_, C_12_) carboxylic acid chlorides to the amides **5b**, **5d‐i** [20]. Subsequent reduction of the carboxamides with LiAlH_4_ led to the target compounds **6b**, **6d‐i** (Scheme 1).

**Scheme 2:**
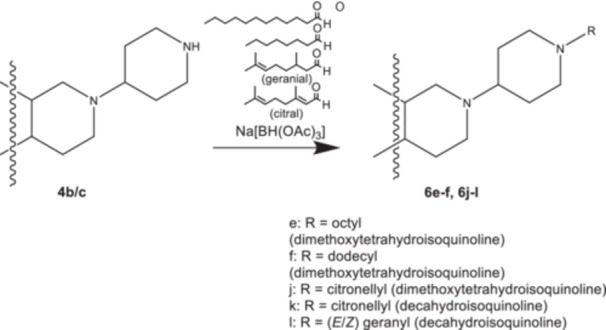
Synthesis of compounds **6e‐f** and **6j‐l**.

In an alternative approach (Scheme 2) the cyclic secondary amines **4b** and **4c** were directly converted into the tertiary amines **6e**, **6 f**, **6j**, **6k**, and **6 l** by reductive alkylation [21, 22] with the corresponding unbranched aliphatic (*n*‐octanal, *n*‐dodecanal) and branched unsaturated aldehydes (geranial, *E*/*Z*‐citral) and sodium triacetoxyborohydride.

**Scheme 3:**
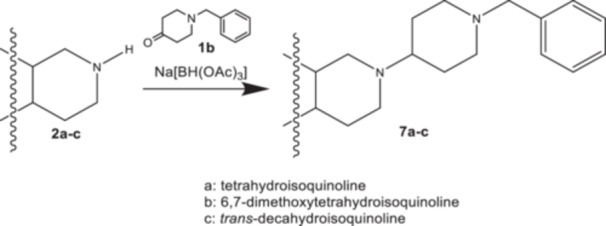
Synthesis of *N*‐benzyl compounds **7a‐c**.

The *N*‐benzylpiperidine derivatives **7a‐c** were obtained from the isoquinolines **2a‐c** and commercially available *N*‐benzyl‐4‐piperidone (**1b**) in a reductive alkylation with sodium triacetoxyborohydride (Scheme 3).

### 2.2 Pharmacology/Biology

The antimycotic activity of all target compounds was evaluated by determining their MIC_100_ in a microdilution [22] assay (Table 1) against *Yarrowia lipolytica*, a non‐pathogenic yeast, which shows a high rate of ergosterol biosynthesis. In previous research we found a good correlation between MIC values against *Yarrowia lipolytica* and pathogenic *Candida* species [21, 22].

**Table 1:** MIC_100_ values against *Yarrowia lipolytica* [23]. (nt: not tested).

compound	MIC_100_ [µg/mL]		compound	MIC_100_ [µg/mL]
**3a**	> 100		**6b**	100
**3b**	> 100		**6 d**	> 100
**3c**	> 100		**6e**	1.6 [4.1 µM]
**4a**	nt		**6 f**	3.1 [7.0 µM]
**4b**	> 100		**6 g**	> 100
**4c**	> 100		**6 h**	3.1 [7.9 µM]
**5b**	> 100		**6i**	1.6 [4.1 µM]
**5 d**	> 100		**6j**	3.1 [7.5 µM]
**5e**	> 100		**6k**	6.3 [17.5 µM]
**5 f**	> 100		**6 l** (*E/Z*)	12.5 [34.9 µM]
**5 g**	> 100		**7a**	> 100
**5 h**	> 100		**7b**	> 100
**5i**	> 100		**7c**	> 100
Clotrimazole	0.4 [1.2 microM]			

The precursors of type **4** and the *N*‐acylpiperidine intermediates of type **5** were inactive, whereas many of the *N*‐alkypiperidines of type **6** showed significant antifungal activity in a concentration range from 1.6 to 12.5 µg/mL. The activities of compounds with remarkable MIC_100_ values in this pre‐screening were further evaluated against human pathogenic yeasts and molds, including also type **6** and type **7** compounds with no activity against *Yarrowia* as (1) control agents and (2) to rule out species‐specific antifungal activity.

Of all test substances, antifungal activity was mainly observed against *Candida* species (Table 2). The compounds **6i** and **6f** with the *n*‐dodecyl side chain showed the highest activities against *Candida* isolates, with MIC_90_ values between 2 and 16 µg/mL, and with the lowest MIC observed for **6f** against *C. krusei*, which is especially noteworthy as this MIC is lower than for fluconazole (FLZ). The branched long‐chain analogues **6k**/**6l** derived from decahydroisoquinoline showed good activity against two *Candida* strains as well, and also against *R. arrhizus*, a member of the Mucorales, which is a group of fungi usually exhibiting an extremely wide resistance profile. These results correspond to earlier results with tetrahydroisoquinoline and imidazole derivatives, in which unbranched alkyl side chains with 10–12 carbon atoms also showed the highest activities [13, 17]. Against the molds, only **6i**, **6k**, and **6l** resulted in a growth inhibition at concentrations of 16–32 µg/mL. In all other cases, no MIC could be determined at the concentrations tested. The broadest antifungal activity was observed for compound **6i**, resulting in growth inhibition at variable concentrations against all species tested, except against Mucorales.

**Table 2:** Minimal inhibitory concentration (MIC_90_) causing 90% growth inhibition of 3 pathogenic yeast and 5 mold isolates of selected synthesized substances with high activity against *Yarrowia lipolytica*. All substances were dissolved in DMSO and diluted in RPMI 1640 medium, containing 2% glucose. All assays were carried out in duplicate. Values represent µg/mL and fluconazole (FLZ) was included as a control agent [24].

Species	6 d	6e	6 f	6 g	6 h	6i	6j	6k	6 l (*E/Z*)	7a	7b	FLZ
*C. albicans*	> 32	32	4	> 32	> 32	4‐8	> 32	16	32	> 32	> 32	0.25
*C. glabrata*	> 32	> 32	4‐8	> 32	> 32	16	> 32	> 32	> 32	> 32	> 32	16
*C. krusei*	> 32	8	2	> 32	8	4‐8	32	8	4	> 32	> 32	8
*A. fumigatus* (A65)	> 32	> 32	> 32	> 32	> 32	32	> 32	> 32	> 32	> 32	> 32	32
*A. fumigatus* (R16)	> 32	> 32	> 32	> 32	> 32	32	> 32	> 32	> 32	> 32	> 32	> 32
*A. fumigatus* (R7)	> 32	> 32	> 32	> 32	> 32	32	> 32	> 32	> 32	> 32	> 32	16
*R. microsporus*	> 32	> 32	> 32	> 32	> 32	> 32	> 32	> 32	> 32	> 32	> 32	> 32
*R. arrhizus (oryzae)*	> 32	> 32	> 32	> 32	32	> 32	> 32	16	16	> 32	> 32	> 32

The cytotoxicity of the active compounds was checked in an MTT assay against human HL 60 cells [25]. The most active compounds **6f** and **6i** showed measurable cytotoxic effects with IC_50_ values of 6 and 23 µg/mL, respectively (Table 3). Although, this corresponds to the MIC range that would be needed in treatment, the values are within the range observed for commonly used antimycotics such as amorolfine and posaconazole. All active compounds were evaluated for their biopharmaceutical behavior and drug‐likeness using the online program SwissADME [26] and should show oral bioavailability according to the Lipinski rules of five [27].

**Table 3:** Determination of cytotoxicity against HL 60 cell line.

Compound	IC_50_ [µg/mL]	Compound	IC_50_ [µg/mL]
**6d**	> 100	**6j**	75
**6e**	> 100	**6k**	17
**6f**	6	**6l**	26
**6g**	> 100	Posaconazole	5
**6h**	75	Amorolfine	17
**6i**	23		

## 3 Conclusion

In summary, we have investigated the antimycotic potential of our series of 2‐(piperidin‐4‐yl)‐1,2,3,4‐tetrahydroisoquinolines and 2‐(piperidin‐4‐yl)decahydroisoquinolines against yeasts and molds including Mucorales. From the results, we gained some deeper understanding of the structure–activity relationships. The activity of the new substances depends heavily on the presence of a nitrogen atom that can be protonated under physiological conditions and an alkyl side chain with a chain length of more than four carbon atoms.

Notably, the substances **6f** and **6i** showed the highest activity against all three studied clinically relevant *Candida* strains. Furthermore, the compounds with remarkable antimycotic activity showed weak cytotoxic activity against a human leukaemia cell line (HL‐60). Although the two most active substances **6f** and **6i** showed the highest cytotoxicity, these values are in the same range as those of the systemically used posaconazole. Since the substances **6j**, **6h**, **6k**, and **6l** show similar cytotoxicity, but less activity against *Candida*, this also gives hope for a selective antimycotic effect.

Summing up, the optimized 2‐(*N*‐alkylpiperidin‐4‐yl)‐1,2,3,4‐tetrahydroisoquinolines and 2‐(N‐alkylpiperidin‐4‐yl)decahydro‐isoquinolines are promising structures for development of novel antimycotics against candidiasis.

## 4 Experimental

### 4.1 Chemistry

#### 4.1.1 General

All solvents used were of HPLC grade or p.a. grade and/or purified according to standard procedures. Chemical reagents were purchased from Sigma Aldrich (Schnelldorf, Germany) and Acros (Geel, Belgium). IR spectra: Jasco FT/IR 4600 series (KBr pellet method or ATR technique); MS: Hewlett Packard MS‐Engine, electron ionization (EI) 70 eV, chemical ionization (CI) with CH_4_ (300 eV); MS spectra: Thermo Q Exactive GC Orbitrap or Finnigan MAT 95 spectrometer, HR‐ESI‐MS spectra: Thermo Finnigan LTQ FT. NMR spectra (see the Supporting Information): Avance III HD 400 MHz Bruker BioSpin (^1^H: 400 MHz, ^13^C: 100 MHz); 500 MHz Avance III HD 500 MHz Bruker BioSpin (^1^H: 500 MHz, ^13^C: 125 MHz); All measurements were taken in deuterated solvents. Chemical shift referencing by tetramethylsilane (TMS) as internal standard or using the solvent signal for calibration. Melting points: Büchi Melting Point B‐540 (not corrected); flash column chromatography (FCC): silica gel 60 (230–400 mesh, E. Merck, Darmstadt). The purities were determined by HPLC. HPLC conditions: System: Shimadzu LC 20 (Prominence), with UV‐ detector, 210 nm/230 nm/254 nm, Column: Phenomenex Kinex 5 µ Biphenyl 100 A, 3 μm, 4.6 mm × 100 mm, 30°C. Mobile phase: acetonitrile/water (1.0% acetic acid) 60:40 (method I) or column: Phenomenex Synergy 4 µ Polar‐RP 80 A, 4.6 mm × 100 mm, 40°C. Mobile phase: methanol/water 90:10 (method II). Mode: isocratic system, flow rare: 1.0 mL/min, concentration: 2–5 mg/mL, injection: 1 µL. The InChI codes of the investigated compounds, together with some biological activity data, are provided as Supporting Information.

#### 4.1.2 General Procedures

General procedure I (reductive amination): The aldehyde or ketone (1.0 eq.) and the amine (1.0–1.4 eq.) were dissolved in 40 mL dry THF. After adding sodium triacetoxyborohydride (2.9 eq.) the suspension was stirred under N_2_ atmosphere for 12 h at room temperature. Then it was quenched with 30 mL saturated aqueous sodium bicarbonate solution for 10 min and extracted with ethyl acetate (3 × 30 mL). The combined organic layers were dried over sodium sulfate and the solvent was removed. The residue was purified by flash column chromatography (isohexane/ethyl acetate 2:8; 0.1 triethylamine or ethyl acetate/isohexane 1:1).

General procedure II (cleavage of the *tert*‐butyloxycarbonyl (Boc) protective group): 20 mL of diethyl ether and 14.0 eq. of methanol were cooled in an ice bath. Then acetyl chloride (9.0 eq.) was added cautiously drop by drop and the solution was stirred for 1 h while still cooling. The Boc protected amine (1.0 eq.) dissolved in 10 mL dry diethyl ether was added and the mixture was stirred for 24 h at room temperature. Subsequently 30 mL of 2 M sodium hydroxide solution were added and the mixture was extracted with ethyl acetate (3 × 30 mL). The combined organic layers were washed with 60 mL distilled water, dried over anhydrous sodium sulfate and the solvent was evaporated. The residue was dried in high vacuum and processed without further cleaning.

General procedure III (preparation of the amides): The secondary amine (1.0 eq.) was dissolved in 10 mL toluene, 1.2 eq. to 1.5 eq. of the carboxylic acid chloride and 1.5–2.0 g (14.8–19.8 mmol) triethylamine were added. The mixture was left stirring overnight at room temperature. Then the solvent was evaporated and the residue was dissolved in 20 mL 2 M aqueous sodium hydroxide solution. The mixture was extracted with ethyl acetate (3 × 20 mL). The combined organic layers were dried over sodium sulfate and the solvent was removed in vacuo. The purification of the residue was performed through flash column chromatography (isohexane/ethyl acetate 2:8; 0.1 triethylamine).

General procedure IV (reduction of the amides): The amide (1.0 eq.) was dissolved in 20 mL of freshly distilled dry tetrahydrofuran and 2.0 eq. to 3.5 eq. of LiAlH_4_ were added. The suspension was refluxed for 2 h. Then it was hydrolyzed cautiously with distilled water and 20 mL of aqueous 2 M sodium hydroxide solution were added, followed by extraction with ethyl acetate (3 × 20 mL). The combined organic layers were dried over sodium sulfate and the solvent was removed. The residue was purified by flash column chromatography (isohexane/ethyl acetate 1:1; 0.1 triethylamine).

#### 4.1.3 Compound Characterization

*tert*‐Butyl 4‐(3,4‐dihydroisoquinolin‐2(1*H*)‐yl)piperidine‐1‐carboxylate (3a): The compound was prepared according to General procedure I from 995 mg (5.0 mmol) of *tert*‐butyl 4‐oxopiperidine‐1‐carboxylate (**1a**) and 931 mg (7.0 mmol) of 1,2,3,4‐tetrahydroisoquinoline (**2a**) to give 948 mg (60%) of 3a as a pale brown oil. ^1^H NMR (400 MHz, chloroform‐*d*) δ 7.24–7.06 (m, 3 H, 3 arom. CH), 7.04–6.97 (m, 1 H, arom. CH), 4.32–4.04 (m, 2 H, 2 CH_2_), 3.79 (s, 2 H, CH_2_), 2.95–2.82 (m, 4 H, 2 CH_2_), 2.80–2.66 (m, 2 H, 2 CH_2_), 2.61 (tt, *J* = 11.4, 3.6 Hz, 1 H, CH), 1.94–1.81 (m, 2 H, 2 CH_2_), 1.64–1.49 (m, 2 H, 2 CH_2_), 1.47 (s, 9 H, 3 CH_3_). ^13^C NMR (100 MHz, chloroform‐*d*) δ 154.76 (CO), 134.47 (quat. C), 134.41 (quat. C), 128.72 (arom. CH), 126.73 (arom. CH), 126.09 (arom. CH), 125.60 (arom. CH), 79.58 (quat. C), 61.45 (CH), 51.94 (CH_2_), 43.15 (2 CH_2_), 29.54 (CH_2_), 28.47 (3 CH_3_), 28.14 (2 CH_2_). IR (ATR): ν (cm^−1^) = 2976, 2930, 1684, 1647, 1426, 1246, 1156, 1013, 740. MS (EI) *m/z* = 259 (M^+^‐C_4_H_9_, 20), 158 (16), 132 (100). HR‐MS calcd. for C_19_H_27_N_2_O_2_ [M^+^‐H]: 315.2068. Found: 315.2068. HPLC purity: > 99% (method II).

*tert*‐Butyl 4‐(6,7‐dimethoxy‐3,4‐dihydroisoquinolin‐2(1*H*)‐yl)piperidine‐1‐carboxylate (**3b**).

The compound was prepared according to General procedure I from 897 mg (4.5 mmol) of **1a** and 1216 mg (6.3 mmol) of 6,7‐dimethoxy‐1,2,3,4‐tetrahydroisoquinoline (**2b**) (freshly prepared from 1441 mg (6.3 mmol) of the commercially available corresponding hydrochloride) to give 1387 mg (82%) of **3b** as an orange oil. ^1^H NMR (400 MHz, chloroform‐*d*) δ 6.59 (s, 1 H, arom. CH), 6.52 (s, 1 H, arom. CH), 4.31–4.14 (m, 2 H, 2 CH_2_), 3.84 (s, 3 H, OCH_3_), 3.83 (s, 3 H, OCH_3_), 3.70 (s, 2 H, CH_2_), 2.87–2.68 (m, 6 H, 4 CH_2_), 2.59 (tt, *J* = 11.3, 3.6 Hz, 1 H, CH), 1.94–1.82 (m, 2 H, 2 CH_2_), 1.59–1.48 (m, 2 H, 2 CH_2_), 1.46 (s, 9 H, 3 CH_3_). ^13^C NMR (100 MHz, chloroform‐*d*) δ 154.77 (CO), 147.53 (quat. C), 147.41 (quat. C), 128.77 (quat. C), 126.28 (quat. C), 111.41 (arom. CH), 109.61 (arom. CH), 79.48 (quat. C), 61.42 (CH), 55.94 (OCH_3_), 55.91 (OCH_3_), 51.50 (CH_2_), 46.90 (CH_2_), 43.24 (2 CH_2_), 29.10 (CH_2_), 28.47 (3 CH_3_), 28.14 (2 CH_2_). IR (ATR): ν (cm^−1^) = 3465, 2934, 2858, 1692, 1612, 1517, 1464, 1256, 1230, 1171, 1133. MS *m/z* (%) = 375 (M^+^‐1, 2), 319 (M^+^‐C_4_H_12_), 20), 218 (18), 192 (6,7‐dimethoxytetrahydroisoquinoline, 100), 164 (24). HR‐MS calcd. for C_21_H_31_N_2_O_4_ (M^+‐^H): 375.2284. Found: 375.2262. HPLC purity: > 92% (method II).

*tert*‐Butyl 4‐(octahydroisoquinolin‐2(1*H*)‐yl)piperidine‐1‐carboxylate (**3c**): The compound was prepared according to General procedure I from 897 mg (4.5 mmol) of *tert*‐butyl 4‐oxopiperidine‐1‐carboxylate (**1a**), 877 mg (6.3 mmol) of *trans*‐decahydroisoquinoline (**2c**) and 2.76 g (13 mmol) of sodium triacetoxyborohydride to give 915 mg (63%) of **3c** as a brown orange oil. ^1^H NMR (400 MHz, chloroform‐*d*): δ 4.24–4.00 (m, 2 H, 2 CH_2_), 2.95–2.84 (m, 1 H, CH_2_), 2.78–2.52 (m, 3 H, 3 CH_2_), 2.47–2.29 (m, 1 H, CH), 2.18 (t, *J* = 11.2 Hz, 1 H, CH_2_), 1.87–1.65 (m, 6 H, 6 CH_2_), 1.64–1.47 (m, 3 H, 3 CH_2_), 1.47–1.34 (m, 2 H, 2 CH_2_), 1.45 (s, 9 H, 3 CH_3_), 1.33–1.10 (m, 4 H, CH, 3 CH_2_), 1.05–0.77 (m, 3 H, CH, 2 CH_2_). ^13^C NMR (101 MHz, chloroform‐*d*): δ 154.75 (CO), 79.26 (quat. C), 62.42 (CH), 56.12 (CH_2_), 50.08 (CH_2_), 43.60 (2 CH_2_), 42.25 (CH), 42.20 (CH), 33.35 (CH_2_), 32.96 (CH_2_), 30.80 (CH_2_), 28.46 (3 CH_3_), 28.02 (2 CH_2_), 26.49 (CH_2_), 26.10 (CH_2_). IR (ATR): ν (cm^−1^) = 2920, 1697, 1423, 1240, 1168. MS (EI): *m/z* (%) 265 ([M^+^‐C_4_H_9_], 29), 178 (50), 166 (57), 140 (100), 138 (68). HR‐MS: calculated for C_19_H_34_N_2_O_2_ (M^+^): 322.2620. Found: 322.261.

2‐(Piperidin‐4‐yl)‐1,2,3,4‐tetrahydroisoquinoline (4a): The compound was prepared according General Procedure II from 700 mg (2.2 mmol) of **3a** to give 330 mg (69%) of 4a as a pale yellow oil. ^1^H NMR (400 MHz, methanol‐*d*_4_) δ 7.16–7.07 (m, 3 H, 3 arom. CH), 7.07–7.00 (m, 1 H, rom. CH), 3.81 (s, 2 H, CH_2_), 3.25–3.18 (m, 2 H, 2 CH_2_), 2.95–2.89 (m, 2 H, CH_2_), 2.89–2.84 (m, 2 H, CH_2_), 2.66 (td, *J* = 12.2, 2.3 Hz, 2 H, 2 CH_2_), 2.57 (tt, *J* = 11.5, 3.7 Hz, 1 H, CH), 1.99–1.89 (m, 2 H, 2 CH_2_), 1.54 (qd, *J* = 12.1, 4.1 Hz, 2 H, 2 CH_2_). ^13^C NMR (100 MHz, methanol‐*d*_4_) δ 133.74 (2 quat. C), 128.31 (arom. CH), 126.47 (arom. CH), 126.24 (arom. CH), 125.64 (arom. CH), 58.32 (CH), 51.24 (CH_2_), 46.36 (CH_2_), 43.32 (2 CH_2_), 28.40 (CH_2_), 24.74 (2 CH_2_). IR (ATR): ν (cm^−1^) = 3247, 2925, 1645, 1451, 1371, 1147, 1092, 932, 883, 815. MS (EI) *m/z* = 215 (1, M^+^‐H), 132 (100). HR‐MS calcd. for. C_14_H_20_N_2_: 216.1626 Found: 216.1620. HPLC purity: > 94% (method II).

6,7‐Dimethoxy‐2‐(piperidin‐4‐yl)‐1,2,3,4‐tetrahydroisoquinoline (**4b**): The compound was prepared according General procedure II from 970 mg (2.58 mmol) of **3b** to give 367 mg (52%) of **4b** as yellow orange oily solid.^1^H NMR (500 MHz, chloroform‐*d*) δ 6.59 (s, 1 H, arom. CH), 6.52 (s, 1 H, arom. CH), 3.84 (s, 3 H, OCH_3_), 3.83 (s, 3 H, 3 OCH_3_), 3.71 (s, 2 H, CH_2_), 3.28–3.21 (m, 2 H, 2 CH_2_), 2.86–2.75 (m, 4 H, 2 CH_2_), 2.69 (td, *J* = 12.2, 2.5 Hz, 2 H, 2 CH_2_), 2.00–1.90 (m, 2 H, 2 CH_2_), 1.70–1.57 (m, 2 H, 2 CH_2_). ^13^C NMR (100 MHz, chloroform‐*d*) δ 147.44 (quat. C), 147.16 (quat. C), 126.94 (quat. C), 126.42 (quat. C), 111.34 (arom. CH), 109.57 (arom. CH), 61.32 (CH), 55.93 (OCH_3_), 55.89 (OCH_3_), 51.45 (CH_2_), 46.72 (CH_2_), 45.99 (2 CH_2_), 29.71 (2 CH_2_), 28.20 (CH_2_). IR (ATR): ν (cm^−1^) = 2929, 1685, 1427, 1131, 1254. MS *m/z* (%) = 232 (4), 218 (8), 192 (100). HR‐MS calcd. for C_16_H_23_N_2_O_2_ (M^+^‐H): 275.1760. Found: 275.1747. HPLC purity: > 93% (method II).

(±)‐2‐(Piperidin‐4‐yl)decahydroisoquinoline (**4c**): The compound was prepared according General procedure II from 800 mg (2.48 mmol) of **3c** to give 327 mg (59%) of **4c** as brown oily solid. ^1^H NMR (400 MHz, chloroform‐*d*) δ 3.13 (m, 2 H, 2 CH_2_), 2.98–2.86 (m, 1 H, CH_2_), 2.82–2.70 (m, 1 H, CH_2_), 2.57 (t, *J* = 12.3 Hz, 2 H, CH_2_), 2.41–2.28 (m, 1 H, CH), 2.23–2.11 (m, 1 H, CH_2_), 1.88–0.75 (m, 18 H, 2 CH, 8 CH_2_, NH). ^13^C NMR (100 MHz, chloroform‐*d*) δ 62.67 (CH), 56.12 (CH_2_), 49.86 (CH_2_), 46.60 (2 CH_2_), 42.29 (2 CH), 33.42 (CH_2_), 33.01 (CH_2_), 30.84 (CH_2_), 29.64 (2 CH_2_), 26.51 (CH_2_), 26.12 (CH_2_). IR (ATR): ν (cm^−1^) = 2919, 2853, 2797, 1445, 1104. MS (EI) *m/z* (%) = 222 (M^+^, 28), 178 (56), 166 (62), 140 (100), 138 (79) 83 (48). HR‐MS calcd. for. C_14_H_26_N_2_: 222.2096. Found: 222.2090.

1‐[4‐(3,4‐Dihydroisoquinolin‐2(1*H*)‐yl)piperidin‐1‐yl]octan‐1‐one (**5b**): The compound was prepared according to General procedure III from 1.08 g (5.0 mmol) of **4a**, 972 mg (6.0 mmol) of *n*‐octanoyl chloride and to give 941 mg (55%) of **5b** as a yellow oily solid. ^1^H NMR (400 MHz, chloroform‐*d*) δ 7.15–7.06 (m, 3 H, 3 arom. CH), 7.05–6.98 (m, 1 H, arom. CH), 4.76–4.63 (m, 1 H, CH_2_), 4.00–3.88 (m, 1 H, CH_2_), 3.78 (s, 2 H, CH_2_), 3.11–3.00 (m, 1 H, CH_2_), 2.93–2.86 (m, 2 H, CH_2_), 2.86–2.80 (m, 2 H, CH_2_), 2.74–2.65 (m, 1 H, CH_2_), 2.64–2.54 (m, 1 H, CH), 2.34 (t, *J* = 7.4 Hz, 2 H,CH_2_), 2.02–1.88 (m, 2 H, 2 CH_2_), 1.72–1.47 (m, 4 H, 3 CH_2_), 1.40–1.22 (m, 8 H, 4 CH_2_), 0.87 (t, *J* = 6.6 Hz, 3 H, CH_3_). ^13^C NMR (101 MHz, chloroform‐*d*) δ 171.55 (CO), 134.88 (quat. C), 134.43 (quat. C), 129.92 (arom. CH), 127.46 (arom. CH), 126.13 (arom. CH), 125.63 (arom. CH), 61.39 (CH), 52.01 (CH_2_), 46.82 (CH_2_), 45.14 (CH_2_), 41.14 (CH_2_), 33.48 (CH_2_), 31.74 (CH_2_), 29.55 (CH_2_), 29.51 (CH_2_), 29.18 (CH_2_), 29.12 (CH_2_), 27.89 (CH_2_), 25.50 (CH_2_), 22.63 (CH_2_), 14.09 (CH_3_). MS (EI) *m/z* = 342 (2, M^+^), 341 (4), (340 (6), 172 (19), 132 (100). HR‐MS calcd. for. C_22_H_34_N_2_O: 342.2671: Found: 342.2665. HPLC purity: > 95% (method II).

1‐[4‐(6,7‐Dimethoxy‐3,4‐dihydroisoquinolin‐2(1*H*)‐yl)‐piperidin‐1‐yl]butan‐1‐one (**5d**): The compound was prepared according to General procedure III from 190 mg (0.689 mmol) of **4b**, 110 mg (1.03 mmol) of *n*‐butanoyl chloride and 1.82 g (18.0 mmol) of triethylamine to give 143 mg (60%) of **5d** as a yellow oily solid. ^1^H NMR (400 MHz, chloroform‐*d*): δ 6.59 (s, 1H, CH), 6.52 (s, 1 H, CH), 4.74–4.66 (m, 1 H, CH_2_), 3.95–3.91 (m, 1 H, CH_2_), 3.84 (d, *J* = 2.5 Hz, 6H, 2 CH_3_), 3.71 (s, 2H, CH_2_), 3.10–3.01 (m, 1H, CH_2_), 2.81 (s, 4 H, 2 CH_2_), 2.72–2.64 (m, 1H, CH), 2.64–2.56 (m, 1 H, CH_2_), 2.36–2.29 (m, 2 H, CH_2_), 1.98–1.91 (m, 2 H, CH_2_), 1.70–1.63 (m, 2 H, CH_2_), 1.57–1.52 (m, 2 H, CH_2_), 1.00–0.95 (m, 3 H, CH_3_). ^13^C NMR (101 MHz, chloroform‐*d*): δ 171.33 (CO), 147.54 (quat. C), 147.22 (quat. C), 126.62 (quat. C), 126.28 (quat. C), 111.39 (arom. CH), 109.49 (arom. CH), 61.28 (CH), 55.93 (CH_3_), 55.90 (CH_3_), 51.51 (CH_2_), 46.91 (CH_2_), 45.08 (CH_2_), 41.08 (CH_2_), 35.32 (CH_2_), 29.19 (CH_2_), 29.05 (CH_2_), 27.94 (CH_2_), 18.85 (CH_2_), 14.04 (CH_2_). IR (ATR): ν (cm^−1^) = 2929, 1634, 1516, 1448, 1256, 1224, 1131. MS (EI): *m/z* (%) 192 (100), 164 (52), 71 (24), 42 (70). HR‐MS: calculated for C_20_H_29_N_2_O_3_ (M^+^‐H): 345.2178. Found: 345.2165. HPLC purity: > 96%.

1‐[4‐(6,7‐Dimethoxy‐3,4‐dihydroisoquinolin‐2(1*H*)‐yl)‐piperidin‐1‐yl]‐octan‐1‐one (**5e**): The compound was prepared according to General procedure III from 553 mg (2.0 mmol) of **4b**, 390 mg (2.4 mmol) of *n*‐octanoyl chloride and 2.02 g (20.0 mmol) of triethylamine to give 167 mg (21%) of **5e** as yellow oily solid. ^1^H NMR (400 MHz, chloroform‐*d*): δ 6.58 (s, 1 H, arom. CH), 6.52 (s, 1 H, arom. CH), 4.74–4.54 (m, 1 H, CH_2_), 4.02–3.86 (m, 1 H, CH_2_), 3.81 (s, 3 H, OCH_3_), 3.81 (s, 3 H, OCH_3_), 3.69 (s, 2 H, CH_2_), 3.04 (t, *J* = 12.8 Hz, 1 H, CH), 2.79 (s, 4 H, 2 CH_2_), 2.73–2.53 (m, 2 H, CH_2_, CH), 2.33 (t, *J* = 7.1 Hz, 2 H, CH_2_), 1.99–1.86 (m, 2 H, 2 CH_2_), 1.62 (h, *J* = 5.8, 4.7 Hz, 2 H, 2 CH_2_), 1.58– (m, 2 H, CH_2_), 1.39–1.21 (m, 8 H, 4 CH_2_), 0.87 (t, *J* = 6.8 Hz, 3 H, CH_3_). ^13^C NMR (100 MHz, chloroform‐*d*): δ 171.26 (CO), 147.41 (quat. C), 147.12 (quat. C), 126.58 (quat. C), 126.21 (quat. C), 111.31 (arom. CH), 109.52 (arom. CH), 61.13 (CH), 55.78 (OCH_3_), 55.75 (OCH_3_), 51.40 (CH_2_), 46.79 (CH_2_), 44.92 (CH_2_), 40.93 (CH_2_), 33.24 (CH_2_), 31.61 (CH_2_), 29.34 (CH_2_), 29.06 (CH_2_), 29.00 (CH_2_), 28.97 (CH_2_), 27.86 (CH_2_), 25.35 (CH_2_), 22.50 (CH_2_), 14.01 (CH_3_). MS (EI): *m/z* (%) = 401 ([M^+^‐1]; 2), 232 (8), 192 (100). HR‐MS: calculated for C_24_H_38_N_2_O_3_: 402.2882. Found: 402.2878. HPLC purity: > 90% (method II).

1‐[4‐(6,7‐Dimethoxy‐3,4‐dihydroisoquinolin‐2(1*H*)‐yl)piperidin‐1‐yl]dodecan‐1‐one (**5f**): The compound was prepared according to General procedure III from 368 mg (1.33 mmol) of **4b**, 436 mg (2.0 mmol) of *n*‐dodecanoyl chloride and 1452 mg (14.3 mmol) of triethylamine to give 192 mg (32%) of **5f** as pale yellow oily solid. ^1^H NMR (400 MHz, chloroform‐*d*): δ 6.59 (s, 1 H, arom. CH), 6.52 (s, 1 H, arom. CH), 4.75–4.60 (m, 1 H, CH_2_), 3.98–3.90 (m, 1 H, CH_2_), 3.83 (s, 3 H, OCH_3_), 3.82 (s, 3 H, OCH_3_), 3.71 (s, 2 H, CH_2_), 3.10–3.00 (m, 1 H, CH_2_), 2.81 (s, 4 H, 2 CH_2_), 2.72–2.64 (m, 1 H, CH), 2.64–2.55 (m, 1 H, CH_2_), 2.36–2.31 (m, 2 H, CH_2_), 2.00–1.90 (m, 2 H, CH_2_), 1.66–1.58 (m, 2 H, CH_2_), 1.57–1.48 (m, 2 H, CH_2_), 1.35–1.21 (m, 18 H, 9 CH_2_), 0.88 (t, *J* = 6.7 Hz, 3 H, CH_3_). ^13^C NMR (101 MHz, chloroform‐*d*): δ 171.56 (CO), 147.58 (quat. C), 147.27 (quat. C), 126.61 (quat. C), 126.29 (quat. C), 111.40 (arom. CH), 109.59 (arom. CH), 61.32 (CH), 55.95 (OCH_3_), 55.92 (OCH_3_), 51.54 (CH_2_), 46.92 (CH_2_), 45.13 (CH_2_), 41.12 (2 CH_2_), 33.49 (CH_2_), 31.92 (CH_2_), 29.64 (CH_2_), 29.63 (CH_2_), 29.56 (CH_2_), 29.54 (CH_2_), 29.47 (CH_2_), 29.35 (CH_2_), 27.93 (2 CH_2_), 25.50 (CH_2_), 22.69 (CH_2_), 14.13 (CH_3_). IR (ATR): ν (cm^‐1^) = 2917, 2850, 1640, 1521, 1467, 1255, 1220, 1132. MS (EI): *m/z* (%) 458 ([M^+^]; 5), 232 (18), 192 (100), 164 (11). HR‐MS: calculated for C_28_H_46_N_2_O_3_ (M^+^): 458.3508. Found: 458.3423. HPLC purity: > 96% (method II).

(±)‐1‐[4‐(Octahydroisoquinolin‐2(1*H*)‐yl)‐piperidin‐1‐yl]‐butan‐1‐one (**5g**): The compound was prepared according to General procedure III from 315 mg (1.42 mmol) of **4c**, 226 mg (2.12 mmol) of *n*‐butanoyl chloride and 1.82 g (18.0 mmol) of triethylamine to give 252 mg (61%) of **5g** as an orange oily solid. ^1^H NMR (400 MHz, chloroform‐*d*): δ 4.72–4.63 (m, 1 H, CH_2_), 3.90 (d, *J* = 13.7 Hz, 1 H, CH_2_), 3.03–2.93 (m, 1 H, CH_2_), 2.90 (d, *J* = 11.0 Hz, 1 H, CH_2_), 2.77–2.69 (m, 1 H, CH_2_), 2.59–2.42 (m, 2 H, CH, CH_2_), 2.36–2.26 (m, 3 H, CH, CH_2_), 2.23–2.12 (m, 1 H, CH_2_), 1.88–1.78 (m, 3 H, 2 CH_2_), 1.73–1.69 (m, 2 H, 2 CH_2_), 1.68–1.61 (m, 4 H, 3 CH_2_), 1.61–1.35 (m, 5 H, 4 CH_2_), 1.27–1.25 (m, 2 H, 2 CH_2_), 0.99– 0.83 (m, 7 H, CH, 3 CH_2_, CH_3_). ^13^C NMR (101 MHz, chloroform‐*d*): δ 171.29 (CO), 62.71 (CH), 55.94 (CH_2_), 50.49 (CH_2_), 49.24 (CH_2_), 41.54 (CH), 40.93 (CH_2_), 40.69 (CH), 35.36 (CH_2_), 32.42 (CH_2_), 31.86 (CH_2_), 28.42 (CH_2_), 28.25 (CH_2_), 26.77 (CH_2_), 26.21 (CH_2_), 25.70 (CH_2_), 18.87 (CH_2_), 14.05 (CH_3_). IR (ATR): ν (cm^−1^) = 2918, 2851, 1643. MS (EI): *m/z* (%) 292 ([M^+^]; 12), 221 (28), 178 (100), 164 (24), 138 (15). HR‐MS: calculated for C_18_H_32_N_2_O (M^+^): 292.2515. Found: 292.2509.

(±)‐1‐[4‐(Octahydroisoquinolin‐2(1*H*)‐yl)piperidin‐1‐yl]octan‐1‐one (**5h**): The compound was prepared according to General procedure III from 334 mg (2.0 mmol) of 4c, 293 mg (1.8 mmol) of *n*‐octanoyl chloride and 1.52 g (15.0 mmol) of triethylamine to give 375 mg (54%) of **5h** as a yellow oily solid. ^1^H NMR (400 MHz, chloroform‐*d*) δ 4.76–4.60 (m, 1 H, CH_2_), 3.98–3.83 (m, 1 H, CH_2_), 3.03–2.93 (m, 1 H, CH_2_), 2.93–2.85 (m, 1 H, CH), 2.78–2.69 (m, 1 H, CH_2_), 2.54–2.42 (m, 2 H, CH_2_), 2.31 (t, *J* = 7.3 Hz, 2 H, CH_2_), 2.25–2.11 (m, 1 H, CH_2_), 1.93–1.36 (m, 13 H, 7 CH_2_), 1.36–1.12 (m, 12 H, 5 CH_2_, 2 CH), 1.08–0.77 (m, 5 H, 2 CH_2_, CH_3_). ^13^C NMR (100 MHz, chloroform‐*d*) δ 171.50 (CO), 62.34 (CH), 56.46 (CH_2_), 55.92 (CH_2_), 50.39 (CH_2_), 49.86 (CH_2_), 45.37 (2 CH_2_), 42.24 (CH), 42.17 (CH), 41.35 (CH_2_), 33.47 (CH_2_), 33.34 (CH_2_), 32.95 (CH_2_), 31.73 (CH_2_), 30.78 (CH_2_), 29.50 (CH_2_), 29.11 (2 CH_2_), 26.47 (CH_2_), 26.08 (CH_2_), 25.50 (CH_2_), 22.63 (CH_2_), 14.09 (CH_3_). MS (EI) *m/z* (%) = 348 (M^+^, 14), 221 (62), 178 (100), 164(38), 138 (24). HR‐MS calcd. for. C_22_H_40_N_2_O: 348.3141. Found: 348.3138.

(±)‐1‐[4‐(Octahydroisoquinolin‐2(1*H*)‐yl)‐piperidin‐1‐yl]‐dodecan‐1‐one (**5i**): The compound was prepared according to General procedure III from 251 mg (1.13 mmol) of **4c** and 371 mg (1.69 mmol) of *n*‐dodecanoyl chloride to give 268 mg (59%) of 5i as a yellow solid. ^1^H NMR (400 MHz, chloroform‐*d*): δ 4.72–4.62 (m, 1 H, CH_2_), 3.92–3.85 (m, 1 H, CH_2_), 3.02–2.93 (m, 1 H, CH_2_), 2.93–2.86 (m, 1H, CH_2_), 2.75–2.69 (m, 1 H, CH_2_), 2.54–2.42 (m, 2 H, CH, CH_2_), 2.34–2.27 (m, 2 H, 2 CH_2_), 2.22–2.11 (m, 1 H, CH_2_), 1.91–1.78 (m, 3 H, 3 CH_2_), 1.73–1.68 (m, 2 H, 2 CH_2_), 1.63–1.58 (m, 3 H, 3 CH_2_), 1.56–1.48 (m, 2 H, 2 CH_2_), 1.46–1.38 (m, 2 H, CH_2_), 1.30–1.21 (m, 19 H, 12 CH_2_), 1.01–0.92 (m, 2 H, 2 CH_2_), 0.91–0.83 (m, 5 H, 2 CH, CH_3_). ^13^C NMR (101 MHz, chloroform‐*d*): δ 171.50 (CO), 62.36 (CH), 56.46 (CH_2_), 49.85 (CH_2_), 45.37 (CH_2_), 42.23 (CH), 42.16 (CH), 41.34 (CH_2_), 33.49 (CH_2_), 33.32 (CH_2_), 32.94 (CH_2_), 30.78 (CH_2_), 29.64 (CH_2_), 29.56 (CH_2_), 29.54 (CH_2_), 29.47 (CH_2_), 29.36 (CH_2_), 29.11 (CH_2_), 28.97 (CH_2_), 27.72 (CH_2_), 27.59 (CH_2_), 26.47 (CH_2_), 26.08 (CH_2_), 25.51 (CH_2_), 22.70 (CH_2_), 14.15 (CH_3_). IR (ATR): ν (cm^−1^) = 2918, 2851, 1640, 1618, 1089. MS (EI): *m/z* (%) 404 ([M^+^]; 8), 221 (36), 178 (100), 164 (29), 138 (24). HR‐MS: calculated for C_26_H_48_N_2_O (M^+^): 404.3767. Found: 404.3760.

2‐(1‐Octylpiperidin‐4‐yl)‐1,2,3,4‐tetrahydroisoquinoline (**6b**): The compound was prepared according to General procedure IV from 325 mg (0.95 mmol) of **5b** and 126 mg (3.32 mmol) of LiAlH_4_ to give 265 mg (85%) of **5c** as a pale yellow oil. ^1^H NMR (400 MHz, chloroform‐*d*) δ 7.15–7.05 (m, 3 H, 3 arom. CH), 7.04–6.97 (m, 1 H, arom. CH), 3.78 (s, 2 H, CH_2_), 3.03 (dd, *J* = 11.2, 3.6 Hz, 2 H, CH_2_), 2.93–2.86 (m, 2 H, CH_2_), 2.85–2.79 (m, 2 H, CH_2_), 2.48 (ddt, *J* = 11.6, 7.6, 3.9 Hz, 1 H, CH), 2.33–2.28 (m, 2 H, CH_2_), 2.02–1.82 (m, 4 H, 2 CH_2_), 1.70 (qd, *J* = 12.2, 4.0 Hz, 2 H, CH_2_), 1.57–1.45 (m, 2 H, 2 CH_2_), 1.36–1.18 (m, 10 H, 5 CH_2_), 0.87 (t, *J* = 6.9 Hz, 3 H, CH_3_). ^13^C NMR (101 MHz, chloroform‐*d*) δ 135.33 (quat. C), 134.63 (quat. C), 128.70 (arom. CH), 126.73 (arom. CH), 125.95 (arom. CH), 125.48 (arom. CH), 61.75 (CH), 58.89 (CH_2_), 53.47 (2 CH_2_), 52.03 (CH_2_), 46.69 (CH_2_), 31.85 (CH_2_), 29.69 (CH_2_), 29.57 (CH_2_), 29.27 (CH_2_), 28.00 (2 CH_2_), 27.74 (CH_2_), 27.23 (CH_2_), 22.67 (CH_2_), 14.11 (CH_3_). IR (ATR): ν (cm^−1^) = 2922, 2855, 1455, 1361, 1057. MS (EI) *m/z* =) 197 (M^+^ + H ‐C_9_H_10_N, 26), 158 (10), 132 (67), 114 (13), 98 (100). HR‐MS calcd. for C_22_H_35_N_2_ (M^+^‐H): 327.2800. Found: 327.2795. HPLC purity: > 97% (method II).

2‐(1‐Butylpiperidin‐4‐yl)‐6,7‐dimethoxy‐1,2,3,4‐tetrahydroisoquinoline (**6d**): The compound was prepared according to General procedure IV from 130 mg (0.376 mmol) of **5d** and 49.9 mg (1.32 mmol) of LiAlH_4_ to give 63 mg (51%) of 5c as a pale yellow oil. ^1^H NMR (400 MHz, chloroform‐*d*): δ 6.58 (s, 1 H, CH), 6.51 (s, 1 H, CH), 3.93–3.91 (m, 1 H, CH_2_), 3.83 (s, 3 H, CH_3_), 3.82 (s, 3 H, CH_3_), 3.71 (s, 2 H, CH_2_), 3.07–3.01 (m, 2 H, 2 CH_2_), 2.82–2.79 (m, 4 H, 2 CH_2_), 2.53–2.44 (m, 1 H, CH), 2.36–2.31 (m, 2 H, CH_2_), 2.01–1.92 (m, 2 H, CH_2_), 1.92–1.84 (m, 2 H, 2 CH_2_), 1.84–1.79 (m, 1 H, CH_2_), 1.52–1.46 (m, 1 H, CH_2_), 1.28–1.24 (m, 3 H, 2 CH_2_), 0.95–0.90 (m, 3 H, CH_3_). ^13^C NMR (101 MHz, chloroform‐*d*): δ 147.46 (quat. C), 147.18 (quat. C), 127.07 (quat. C), 126.48 (quat. C), 111.42 (arom. CH), 109.64 (arom. CH), 58.49 (CH_2_), 56.09 (CH), 55.91 (2 CH_3_), 53.35 (CH_2_), 53.12 (CH_2_), 51.58 CH_2_), 46.80 (CH_2_), 29.70 (CH_2_), 29.21 (CH_2_), 29.10 (CH_2_), 27.97 (CH_2_), 20.86 (CH_2_), 14.07 (CH_3_). IR (ATR): ν (cm^−1^) = 2928, 1519, 1450, 1275, 1254, 1130. MS (EI): *m/z* (%) 193 (12), 192 (100), 98 (18), 96(27). HR‐MS: calculated for C_20_H_32_N_2_O_2_: 322.2464. Found: 332.2454. HPLC purity: > 87% (method II).

6,7‐Dimethoxy‐2‐(1‐octylpiperidin‐4‐yl)‐1,2,3,4‐tetrahydroisoquinoline (**6e**): The compound was prepared according to General procedure IV from 250 mg (0.62 mmol) of **5e** and 50 mg (1.32 mmol) of LiAlH_4_ to give 108 mg (45%) of **6e** as a pale yellow oil. ^1^H NMR (400 MHz, chloroform‐*d*) δ 6.58 (s, 1 H, arom. CH), 6.51 (s, 1 H, arom. CH), 3.83 (s, 3 H, CH_3_), 3.83 (s, 3 H, CH_3_), 3.70 (s, 2 H, CH_2_), 3.07–2.97 (m, 2 H, 2 CH_2_), 2.86–2.76 (m, 4 H, 2 CH_2_), 2.47 (tt, *J* = 11.5, 3.7 Hz, 1 H, CH), 2.34–2.26 (m, 2 H, CH_2_), 2.03–1.82 (m, 4 H, 4 CH_2_), 1.69 (qd, *J* = 12.1, 3.8 Hz, 2 H, 2 CH_2_), 1.54–1.45 (m, 2 H, CH_2_), 1.35–1.20 (m, 10 H, 5 CH_2_), 0.87 (t, *J* = 6.7 Hz, 3 H, CH_3_). ^13^C NMR (100 MHz, chloroform‐*d*) δ 147.43 (quat. C), 147.15 (quat. C), 127.14 (quat. C), 126.51 (quat. C), 111.41 (arom. CH), 109.63 (arom. CH), 61.73 (CH), 58.89 (CH_2_), 55.93 (OCH_3_), 55.89 (OCH_3_), 53.47 (2 CH_2_), 51.60 (CH_2_), 46.79 (CH_2_), 31.84 (CH_2_), 29.57 (CH_2_), 29.26 (CH_2_), 29.25 (CH_2_), 28.09 (2 CH_2_), 27.74 (CH_2_), 27.23 (CH_2_), 22.67 (CH_2_), 14.15 (CH_3_). IR (ATR): ν (cm^−1^) = 2924, 2848, 1610, 1520, 1470, 1772, 1256, 1229, 1129, 1020. MS (EI) *m/z* (%) = 387 (M^+^‐1, 2), 192 (100), 96 (29). HR‐MS calcd. for. C_24_H_39_N_2_O_2_: 387.3012. Found: 387.3009. HPLC purity: > 99% (method II).

2‐(1‐Dodecylpiperidin‐4‐yl)‐6,7‐dimethoxy‐1,2,3,4‐tetrahydroisoquinoline (**6f**): The compound was prepared according to General procedure IV from 192 mg (0.419 mmol) of **5f** and 32 mg (0.84 mmol) of LiAlH_4_ to give 156 mg (84%) of 6 f as a pale yellow oil. ^1^H NMR (400 MHz, chloroform‐*d*): δ 6.58 (s, 1 H, CH), 6.51 (s, 1 H, CH), 3.83 (d, *J* = 2.1 Hz, 6 H, 2 CH_3_), 3.71 (s, 2 H, CH_2_), 3.07–3.01 (m, 2 H, CH_2_), 2.81 (s, 4 H, 2 CH_2_), 2.52–2.44 (m, 1 H, CH), 2.01–1.93 (m, 2 H, 2 CH_2_), 1.91–1.85 (m, 2 H, 2 CH_2_), 1.76–1.68 (m, 2 H, 2 CH_2_), 1.53–1.46 (m, 2 H, 2 CH_2_), 1.30–1.24 (m, 20 H, 10 CH_2_), 0.90–0.86 (m, 3 H, CH_3_). ^13^C NMR (101 MHz, chloroform‐*d*): δ 147.44(quat. C), 147.16 (quat. C), 127.05 (qua. C), 126.49 (quat. C), 111.39 (CH), 109.61(CH), 61.68 (CH), 55.90 (2 CH_3_), 53.42 (CH_2_), 51.50 (CH_2_), 46.80 (3 CH_2_), 31.93 (CH_2_), 29.68 (CH_2_), 29.65 (CH_2_), 29.63 (CH_2_), 29.60 (CH_2_), 29.36 (CH_2_), 29.19 (CH_2_), 28.47 (2 CH_2_), 27.84 (CH_2_), 27.73 (CH_2_), 27.08 (CH_2_), 22.70 (CH_2_), 14.13 (CH_3_). IR (ATR): ν (cm^−1^) = 2916, 1256, 1229, 1131. MS (EI): *m/z* (%) 444 ([M^+^]; 2), 248 (92), 192 (100), 98 (72), 96 (47). HR‐MS: calculated. for C_28_H_48_N_2_O_2_ (M^+^): 444.3716. Found: 444.3717. HPLC purity: > 99% (method II). In an alternative approach this compound was prepared according to General procedure I from 210 mg (0.76 mmol) of 4b and 136 mg (1.06 mmol) of *n*‐octanal to give 196 mg (66%) of **6f**.

(±)‐2‐(1‐Butylpiperidin‐4‐yl)‐decahydroisoquinoline (**6g**): The compound was prepared according to General procedure IV from 230 mg (0.787 mmol) of **5g** and 105 mg (2.75 mmol) of LiAlH_4_ to give 94 mg (43%) of **6g** as an orange brown oily solid. ^1^H NMR (400 MHz, chloroform‐*d*): δ 3.05–2.93 (m, 3 H, 3 CH_2_), 2.82–2.76 (m, 1 H, CH_2_), 2.44–2.34 (m, 1 H, CH), 2.34–2.28 (m, 2 H, CH_2_), 2.28–2.22 (m, 1 H, CH_2_), 1.97–1.87 (m, 3 H, 3 CH_2_), 1.87–1.80 (m, 2 H, 2 CH_2_), 1.74–1.68 (m, 2 H, CH_2_), 1.68–1.62 (m, 2 H, 2 CH_2_), 1.56–1.43 (m, 4 H, 4 CH_2_), 1.42–1.36 (m, 1 H, CH_2_), 1.36–1.19 (m, 6 H, CH, 5 CH_2_), 1.03–0.94 (m, 1 H, CH_2_), 0.94–0.88 (m, 4 H, CH_2_, CH_3_), 0.88–0.82 (m, 1 H, CH). ^13^C NMR (101 MHz, chloroform‐*d*): δ 62.68 (CH), 58.40 (CH_2_), 55.91 (CH_2_), 53.39 (2 CH_2_), 49.85 (CH_2_), 42.02 (CH), 41.68 (CH), 32.86 (CH_2_), 32.78 (CH_2_), 30.68 (CH_2_), 29.25 (CH_2_), 27.41 (CH_2_), 27.37 (CH_2_), 26.41 (CH_2_), 25.95 (CH_2_), 20.83 (CH_2_), 14.03 (CH_3_). IR (ATR): ν (cm^−1^) = 2917, 2850, 1446. MS (EI): *m/z* (%) 140 (20), 139 (63), 98 (24), 96 (100). HR‐MS: calculated for C_18_H_34_N_2_: 278.2722. Found: 278.2717.

(±)‐2‐(1‐Octylpiperidin‐4‐yl)decahydroisoquinoline (**6h**): The compound was prepared according to General procedure IV from 376 mg (1.08 mmol) of **5h** and 82 mg (2.2 mmol) of LiAlH_4_ to give 330 mg (91%) of **6h** as a pale yellow oil. ^1^H NMR (400 MHz, chloroform‐*d*) δ 3.03–2.93 (m, 2 H, 2 CH_2_), 2.93–2.85 (m, 1 H, CH_2_), 2.76–2.68 (m, 1 H, CH_2_), 2.33–2.23 (m, 3 H, 2 CH_2_), 2.23–2.14 (m, 1 H, CH), 1.94–1.80 (m, 2 H, 2 CH_2_), 1.81–1.66 (m, 4 H, 4 CH_2_), 1.67–1.38 (m, 5 H, 5 CH_2_), 1.40–1.09 (m, 17 H, 2 CH, 8 CH_2_), 1.07–0.75 (m, 3 H, 3 CH_2_), 0.88 (t, *J* = 6.7 Hz, 3 H, CH_3_). ^13^C NMR (100 MHz, chloroform‐*d*) δ 62.65 (CH), 58.92 (CH_2_), 56.21 (CH_2_), 53.71 (2 CH_2_), 49.89 (CH_2_), 42.27 (2 CH), 33.41 (CH_2_), 33.00 (CH_2_), 31.85 (CH_2_), 30.82 (CH_2_), 29.57 (CH_2_), 29.26 (CH_2_), 27.79 (CH_2_), 27.74 (CH_2_), 27.72 (CH_2_), 27.27 (2 CH_2_), 26.52 (CH_2_), 26.12 (CH_2_), 22.67 (CH_2_), 14.11 (CH_3_). IR (ATR): ν (cm^−1^) = 2918, 2851, 1446, 1376, 1090. MS (EI) *m/z* = 333 ([M^+^‐H], 0.7), 195 (26), 152 (20), 124 (20), 96 (100). HR‐MS calcd. for C_22_H_41_N_2_ [M^+^‐H]: 333.3348. Found: 333.3264.

(±)‐2‐(1‐Dodecylpiperidin‐4‐yl)‐decahydroisoquinoline (**6i**): The compound was prepared according to General procedure IV from 268 mg (0.662 mmol) of **5i** and 50 mg (1.32 mmol) of LiAlH_4_ to give 120 mg (46%) of **6i** as a yellow oily solid. ^1^H NMR (400 MHz, chloroform‐*d*): δ 3.02–2.94 (m, 2 H, 2 CH_2_), 2.92–2.86 (m, 1 H, CH_2_), 2.75–2.69 (m, 1 H, CH_2_), 2.29–2.25 (m, 2 H, CH_2_), 2.23–2.16 (m, 1 H, CH), 1.91–1.81 (m, 3 H, 2 CH_2_), 1.79–1.68 (m, 5 H, 3 CH_2_), 1.63–1.43 (m, 8 H, 4 CH_2_), 1.29–1.22 (m, 21 H, 11 CH_2_), 0.88 (t, *J* = 6.7 Hz, 3 H, CH_3_), 1.04–0.80 (m, 3 H, 2 CH, CH_2_). ^13^C NMR (101 MHz, chloroform‐*d*): δ 62.62 (CH), 58.87 (CH_2_), 56.15 (CH_2_), 53.62 (2 CH_2_), 50.31 (CH_2_), 42.22 (CH), 42.12 (CH) 33.31 (CH_2_), 32.95 (CH_2_), 31.93 (CH_2_), 30.80 (CH_2_), 29.68 (CH_2_), 29.65 (CH_2_), 29.63 (CH_2_), 29.60 (2 CH_2_), 29.36 (CH_2_), 27.73 (CH_2_), 27.65 (2 CH_2_), 27.21 (CH_2_), 26.50 (CH_2_), 26.08 (CH_2_), 22.70 (CH_2_), 14.13 (CH_3_). IR (ATR): ν (cm^−1^) = 2919, 2849. MS (EI): *m/z* (%) 390 ([M^+^]; 5), 251 (88), 248 (28), 98 (31), 96 (100). HR‐MS: calculated. for C_26_H_50_N_2_ (M^+^): 390.3974. Found: 390.3966.

(±)‐2‐[1‐(3,7‐Dimethyloct‐6‐en‐1‐yl)piperidin‐4‐yl]‐6,7‐dimethoxy‐1,2,3,4‐tetrahydroisoquinoline (**6j**): The compound was prepared according to General procedure I from 150 mg (0.544 mmol) of **4b**, 117 mg (0.762 mmol) of (±)‐citronellal and 333 mg (1.57 mmol) of sodium triacetoxyborohydride to give 123 mg (55%) of 6j as a light yellow oil. ^1^H NMR (400 MHz, chloroform‐*d*): δ 6.58 (s, 1 H, CH), 6.51 (s, 1 H, CH), 5.13–5.06 (m, 1H, CH), 3.83 (s, 3 H, CH_3_), 3.829 (s, 3 H, CH_3_), 3.71 (s, 2 H, CH_2_), 3.12–3.04 (m, 2 H, 2 CH_2_), 2.81 (s, 4 H, 2 CH_2_), 2.55–2.45 (m, 1 H, CH), 2.45–2.33 (m, 2 H, CH_2_), 2.00 – 1.96 (m, 2 H, 2 CH_2_), 1.93–1.85 (m, 2 H, CH_2_), 1.81–1.72 (m, 2 H, CH_2_), 1.69 (s, 3 H, CH_3_), 1.60 (s, 3 H, CH_3_), 1.57–1.48 (m, 2 H, CH_2_), 1.47–1.45 (m, 1 H, CH), 1.38–1.30 (m, 2 H, 2 CH_2_), 1.28–1.24 (m, 2 H, CH_2_), 0.90 (d, *J* = 6.5 Hz, 3 H, CH_3_). ^13^C NMR (101 MHz, chloroform‐*d*): δ 147.48 (quat. C), 147.20 (quat. C), 131.21 (quat. C), 126.91 (quat. C), 126.43 (quat. C), 124.78 (═CH), 111.40 (arom. CH), 109.62 (arom. CH), 56.70 (CH_2_), 56.05 (CH), 55.94 (CH_3_), 55.90 (CH_3_), 53.24 (CH_2_), 53.07 (CH_2_), 51.47 (CH_2_), 46.78 (CH_2_), 37.19 (CH_2_), 33.69 (CH_2_), 31.25 (CH), 29.11 (CH_2_), 27.64 (CH_2_), 25.72 (CH_3_), 25.47 (CH_2_), 19.69 (CH_3_), 17.66 (CH_3_). IR (ATR): ν (cm^−1^) = 2922, 1610, 1520, 1458, 1256, 1229, 1127. MS (EI): *m/z* (%) 413 ([M^+^‐H]; 0,2), 192 (100), 138 (19), 98 (14), 96 (9). HR‐MS: (M^+^‐H): calculated for C_26_H_41_N_2_O_2_: 413.3246. Found: 413.3167. HPLC purity: > 95% (method II).

(±)‐2‐[1‐(3,7‐Dimethyloct‐6‐en‐1‐yl)‐piperidin‐4‐yl]‐decahydroisoquinoline (**6k**): The compound was prepared according to General procedure I from 280 mg (1.26 mmol) of **4c**, 272 mg (1.76 mmol) of (±)‐citronellal and 771 mg (3.64 mmol) of sodium triacetoxyborohydride to give 427 mg (94%) of **6k** as a brown orange oil. ^1^H NMR (400 MHz, chloroform‐*d*): δ 5.12–5.06 (m, 1 H, CH), 3.72–3.64 (m, 1 H, CH_2_), 3.02–2.96 (m, 2 H, 2 CH_2_), 2.92–2.85 (m, 1 H, CH_2_), 2.75–2.69 (m, 1 H, CH_2_), 2.38–2.24 (m, 4 H, CH, 2 CH_2_), 2.24–2.16 (m, 1 H, CH_2_), 1.99–1.93 (m, 2H, CH_2_), 1.90–1.81 (m, 3 H, 3 CH_2_), 1.79–1.69 (m, 4 H, 2 CH_2_), 1.69–1.67 (m, 3 H, CH_3_), 1.62–1.59 (m, 3 H, CH_3_), 1.51–1.48 (m, 1 H, CH_2_), 1.45–1.37 (m, 2 H, CH_2_), 1.32–1.28 (m, 2 H, 2 CH_2_), 1.26–1.25 (m, 1 H, CH), 1.25–1.22 (m, 1 H, CH_2_), 1.22–1.17 (m, 2 H, CH_2_), 1.17–1.12 (m, 2 H, 2 CH_2_), 1.03– 0.95 (m, 1 H, CH_2_), 0.93–0.86 (m, 6 H, 2 CH, CH_2_, CH_3_). ^13^C NMR (101 MHz, chloroform‐*d*): δ 131.13 (quat. C) 124.86 (═CH), 62.65 (CH), 61.12 (CH_2_), 56.85 (CH_2_), 55.65 (CH_2_), 53.82 (CH_2_), 53.62 (CH_2_), 49.87 (CH_2_), 42.22 (CH), 42.10 (CH), 37.25 (CH_2_), 34.18 (CH_2_), 33.26 (CH_2_), 32.95 (CH_2_), 31.28 (CH), 30.79 (CH_2_), 27.66 (CH_2_), 27.61 (CH_2_), 26.50 (CH_2_), 26.07 (CH_2_), 25.72 (CH_3_), 25.49 (CH_2_), 19.72 (CH_3_), 17.65 (CH_3_). IR (ATR): ν (cm^−1^) = 2918, 2851, 1446, 1376, 1089. MS (EI): *m/z* (%) 360.3503 (22), 221 (52), 178 (27), 138 (33), 136 (73), 98 (36), 96 (100), 70 (28). HR‐MS: calculated for C_24_H_44_N_2_: 360.3504. Found: 360.3503.

2‐{1‐[(2*E*)‐3,7‐Dimethylocta‐2,6‐dien‐1‐yl]piperidin‐4‐yl}‐decahydroisoquinoline (**6l**): The compound was prepared according to General procedure I from 370 mg (1.67 mmol) of **4c**, 355 mg (2.33 mmol) of citral (*E*/*Z*, 33%, 66%) and 1.02 g (4.8 mmol) of sodium triacetoxyborohydride to give 621 mg (100%) of **6l** as an orange brown oily solid (*E*/*Z*‐mixture). The mixture of diastereomers could be separated partially by flash column chromatography to give 19 mg (9%) of pure *E*‐isomer (based on 33% of the total yield of 621 mg): ^1^H NMR (400 MHz, chloroform‐*d, E*): δ 5.29–5.22 (m, 1 H, CH), 5.12–5.06 (m, 1 H, CH), 3.05–2.98 (m, 2 H, 2 CH_2_), 2.96 (d, *J* = 6.9 Hz, 2 H, CH_2_), 2.70–2.58 (m, 2 H, 2 CH_2_), 2.46–2.30 (m, 3 H‚CH, CH_2_), 2.09 (q, *J* = 7.0, 5.9 Hz, 3 H, 2 CH_2_), 2.06–2.02 (m, 3 H, CH_3_), 1.99–1.89 (m, 3 H, 3 CH_2_), 1.86–1.76 (m, 3 H, 3 CH_2_), 1.75–1.71 (m, 2 H, 2 CH_2_), 1.70–1.66 (m, 6 H, 3 CH_2_), 1.63–1.61 (m, 3 H, CH_3_), 1.60 (dd, *J* = 5.2, 1.3 Hz, 4 H, CH, 3 CH_2_), 1.57–1.49 (m, 3 H, CH_3_), 1.21 (m, 1 H, CH). ^13^C NMR (101 MHz, chloroform‐*d*, trans): δ 131.72 (quat. C), 131.55 (quat. C), 124.13 (CH), 123.45 (CH), 62.59 (CH), 56.76 (CH_2_), 55.93 (CH_2_), 55.75 (CH_2_), 53.21 (CH_2_), 49.87 (CH_2_), 39.79 (CH_2_), 39.55 (CH_2_), 34.29 (CH), 32.17 (CH_2_), 30.72 (CH_2_), 27.83 (CH_2_), 27.50 (CH_2_), 26.91 (CH_2_), 26.50 (CH_2_), 26.40 (CH_2_), 25.72 (CH_3_), 25.68 (CH_3_), 23.55 (CH), 17.69 (CH_3_). IR (ATR; *trans*): ν (cm^−1^) = 2920, 2858, 1447, 1376. MS (EI, trans): *m/z* (%): 358 ([M^+^]; 27), 235 (44), 221 (70), 178 (68), 152 (86), 150 (54), 138 (41), 136 (45), 124 (50), 96 (56), 83 (57), 69 (100), 41 (54). HR‐MS (trans): calculated for C_24_H_42_N_2_: 358.3348. Found: 358.3343.

The mixture of diastereomers could be separated partially by flash column chromatography to give 389 mg (95%) *Z*‐isomer (based on 66% of the total yield of 621 mg): ^1^H NMR (400 MHz, chloroform‐*d*, Z): δ 5.29– 5.23 (m, 1 H, CH), 5.10–5.05 (m, 1 H, CH), 3.05–2.98 (m, 2 H, 2 CH_2_), 2.98–2.90 (m, 3 H, 3 CH_2_), 2.79–2.73 (m, 1 H, CH_2_), 2.66–2.59 (m, 1 H, CH_2_), 2.43–2.33 (m, 1 H, CH), 2.31–2.20 (m, 1 H, CH_2_), 2.11–2.06 (m, 1 H, CH_2_), 1.95–1.87 (m, 3 H, 3 CH_2_), 1.81 (d, *J* = 11.9 Hz, 2 H, 2 CH_2_), 1.67 (dd, *J* = 2.5, 1.3 Hz, 4 H, 4 CH_2_), 1.64–1.58 (m, 7 H, CH_2_, 2 CH_3_), 1.55–1.53 (m, 1 H, CH_2_), 1.51 (d, *J* = 4.0 Hz, 1 H, CH_2_), 1.41–1.31 (m, 3 H, 3 CH_2_), 1.28– 1.24 (m, 3 H, CH_3_), 1.21–1.19 (m, 2 H, CH, CH_2_), 0.92 – 0.89 (m, 4 H, CH, 3 CH_2_). ^13^C NMR (101 MHz, chloroform‐*d*, Z): δ 131.77 (quat. C), 131.53 (quat. C), 124.14 (CH), 124.03 (CH), 62.57 (CH), 55.87 (CH_2_), 53.34 (CH_2_), 53.23 (CH_2_), 49.79 (CH_2_), 45.99 (CH_2_), 42.08 (2 CH), 41.11 (CH_2_), 39.78 (CH_2_), 32.82 (CH_2_), 32.15 (CH_2_), 30.71 (CH_2_), 27.24 (CH_2_), 26.44 (CH_2_), 26.39 (CH_2_), 25.98 (CH_2_), 25.71 (CH_3_), 23.95 (CH_3_), 17.35 (CH_2_), 16.35 (CH_3_). IR (ATR; *cis*): ν (cm^−1^) = 2919, 2852,1446, 1376, 732. MS (EI, Z): *m/z* (%): 358 ([M^+^]; 28), 219 (50), 178 (50), 152 (53), 150 (71), 136 (48), 83 (76), 69 (100), 40 (41). HR‐MS (Z): calculated for C_24_H_42_N_2_: 358.3348. Found: 358.3352. HPLC purity: > 97% (method I).

2‐(1‐Benzylpiperidin‐4‐yl)‐1,2,3,4‐tetrahydroisoquinoline (**7a**): The compound was prepared according to General procedure I from 946 mg (5.0 mmol) of *N*‐benzyl‐4‐piperidone (**1a**) and 666 mg (5.0 mmol) of 1,2,3,4‐tetrahydroisoquinoline (**2a**) to give 704 mg (46%) of **7a** as a colourless oil. ^1^H NMR (400 MHz, chloroform‐*d*) δ 7.35–7.19 (m, 5 H, 5 arom. CH), 7.12–6.96 (m, 4 H, 4 arom. CH), 3.77 (s, 2 H, CH_2_), 3.51 (s, 2 H, CH_2_), 3.02–2.92 (m, 2 H, CH_2_), 2.91–2.76 (m, 4 H, 2 CH_2_), 2.53‐2.42 (m, 1 H, CH), 2.06–1.95 (m, 2 H, CH_2_), 1.92–1.80 (m, 2 H, 2 CH_2_), 1.71 (td, *J* = 12.0, 3.5 Hz, 2 H, 2 CH_2_). ^13^C NMR (100 MHz, chloroform‐*d*) δ 138.55 (quat. C), 135.30 (quat. C), 134.61 (quat. C), 129.12 (2 arom. CH), 128.70 (arom. CH), 128.18 (2 arom. CH), 126.95 (arom. CH), 126.73 (arom. CH), 125.97 (arom. CH), 125.50 (arom. CH), 63.07 (CH_2_), 61.66 (CH), 53.27 (2 CH_2_), 52.02 (CH_2_), 46.81 (CH_2_), 29.68 (CH_2_), 28.11 (2 CH_2_). IR (ATR): ν (cm^−1^) = 2937, 2811, 1645, 1602, 1493, 1454, 1359, 1090, 1029. MS (EI) *m/z* = 173 (10), 132 (8), 91 (100). HR‐MS calcd. for. C_21_H_25_N_2_ [M^+^‐1]: 305.2018. Found: 305.2010. HPLC purity: 86% (method I).

2‐(1‐Benzylpiperidin‐4‐yl)‐6,7‐dimethoxy‐1,2,3,4‐tetrahydroisoquinoline (**7b**): The compound was prepared according General procedure I from 946 mg (5.0 mmol) of *N*‐benzyl‐4‐piperidone (**1a**) and (5.0 mmol) of 6,7‐dimethoxy‐1,2,3,4‐tetrahydroisoquinoline (**2b**) to give 1.15 g (63%) of 7b as a white solid. M.P.: 120.8°C. ^1^H NMR (400 MHz, chloroform‐*d*) δ 7.34–7.29 (m, 4 H, 4 arom. CH), 7.28–7.22 (m, 1 H, arom. CH), 6.57 (s, 1H, arom. CH), 6.51 (s, 1 H, arom. CH), 3.83 (s, 3 H, OCH_3_), 3.82 (s, 3 H, OCH_3_), 3.71 (s, 2 H, CH_2_), 3.53 (s, 2 H, CH_2_), 3.06–2.93 (m, 2 H, 2 CH_2_), 2.85–2.79 (m, 4 H, 2 CH_2_), 2.55–2.42 (m, 1 H, CH), 2.09–1.96 (m, 2 H, 2 CH_2_), 1.93–1.81 (m, 2 H, 2 CH_2_), 1.76–1.64 (m, 2 H, 2 CH_2_). ^13^C NMR (100 MHz, chloroform‐*d*) δ 147.46 (quat. C), 147.19 (quat. C), 138.44 (quat. C), 129.15 (2 arom. CH), 128.20 (2 arom. CH), 127.04 (quat. C), 126.99 (arom. CH), 126.47 (quat. C), 111.41 (arom. CH), 109.64 (arom. CH), 63.04 (CH_2_), 61.59 (CH), 55.94 (CH_3_), 55.91 (CH_3_), 53.23 (CH_2_), 51.58 (CH_2_), 46.90 (2 CH_2_), 29.20 (CH_2_), 28.15 (2 CH_2_). IR (ATR): ν (cm^−1^) = 2964, 2938, 2919, 1606, 1520, 1456, 1366, 1256, 1128, 1099, 1016, 858MS (EI) *m/z* = 275 (M^+^‐91, 3), 192 (100), 91 (60). HR‐MS calcd. for. C_23_H_30_N_2_O_2_ [M^+^]: 366.2307. Found: 366.2323. HPLC purity: 95% (method I).

2‐(1‐Benzylpiperidin‐4‐yl)decahydroisoquinoline (**7c**): The compound was prepared according General procedure I from 568 mg (5.0 mmol) of *N*‐benzyl‐4‐peridone (**1a**) and 696 mg (4.2 m mol) of *trans*‐decahydroisoquinoline (**2c**) to give 667 mg (71%) of **7c** as a white solid. M.P.: 61.7°C. ^1^H NMR (400 MHz, chloroform‐*d*) δ 7.36–7.19 (m, 5 H, 5 arom. CH), 3.48 (s, 2 H, CH_2_), 2.97–2.87 (m, 2 H, CH_2_), 2.79–2.70 (m, 2 H, CH_2_), 2.33–2.23 (m, 1 H, CH_2_), 2.22–2.14 (m, 1 H, CH), 1.94 (td, *J* = 11.8, 2.4 Hz, 2 H, CH_2_), 1.83 (t, *J* = 10.8 Hz, 1 H, CH), 1.79–1.66 (m, 4 H, 2 CH_2_), 1.66–1.47 (m, 6 H, 4 CH_2_), 1.37–1.11 (m, 4 H, CH, 3 CH_2_), 1.05–0.74 (m, 2 H, CH, CH_2_). ^13^C NMR (100 MHz, chloroform‐*d*) δ 138.58 (quat. C), 129.10 (2 arom. CH), 128.13 (2 arom. CH), 126.89 (arom. CH), 63.06 (CH_2_), 62.61 (CH), 56.14 (CH_2_), 53.45 (2 CH_2_), 50.05 (CH_2_), 42.20 (CH), 42.12 (CH), 32.95 (CH_2_), 30.80 (CH_2_), 27.86 (2 CH_2_), 27.76 (CH_2_), 26.50 (CH_2_), 26.09 (CH_2_). IR (ATR): ν (cm^−1^) = 2920, 2847, 1649, 1493, 1449, 1367, 1110, 1027, 977. MS (EI) *m/z* = 221 (M^+^‐Bz, 8), 173 (30), 134 (6), 91 (100). HR‐MS calcd. for. C_21_H_32_N_2_: 312.2565. Found: 312.2569. HPLC purity: > 98% (method I).

### 4.2 Pharmacological Assays

Determination of MICs against *Yarrowia lipolytica* (DSMZ, Braunschweig, Germany) was carried out according to lit [22]. *Yarrowia lipolytica* was cultivated in AC‐agar (Sigma Aldrich). Concentration of yeast cells was determined by photometer and adjusted to a turbidity of 0.5 according to McFarland standard at 600 nm. All substances were dissolved in DMSO or methanol.

Determination of MICs (minimal inhibitory concentration) against *Candida* species was carried out according to the European Committee of Antifungal Susceptibility Testing [23, 24]. Concentration of yeast cells was determined by photometer and adjusted to a turbidity of 0.5 according to McFarland standard. MIC_90_ was determined by microdilution plate reader (Sunrise Tecan) at 450 nm, after 24 h incubation at 37°C. MIC_90_ was defined as the minimal concentration of chemical causing 90% growth inhibition, respectively. Growth inhibition of molds was determined by eye and microscopically, following EUCAST guidelines. All substances were dissolved in DMSO and diluted in RPMI 1640 medium, containing 2% glucose. All assays were carried out twice.

#### 4.2.1 Cytotoxicity Assay

HL‐60 cells (human leukemia cells, DSM No. ACC3) were obtained from DSMZ (German Collection of Microorganisms and Cell Cultures, Braunschweig, Germany) and cultivated in RPMI 1640 medium with 10% fetal bovine serum (FBS), both from PAA Laboratories, Cölbe, Germany) without the addition of antibiotics at 37°C in a humidified atmosphere containing 5% CO_2_. The assay was performed according to lit [28].

## Conflicts of Interest

The authors declare no conflicts of interest.

## Supporting information

ArchPharm SupplMat NMR FB.

Krauss et al InChI.

## Data Availability

The data that supports the findings of this study are available in the supporting material of this article.
