# Supplementary material for: Synthesis and Biological Evaluation of Novel 2‐(Piperidin‐4‐yl)‐1,2,3,4‐tetrahydroisoquinoline and 2‐(Piperidin‐4‐yl)decahydroisoquinoline Antimycotics
Source: Arch Pharm (Weinheim). 2025 Oct 16;358(10):e70128. doi: 10.1002/ardp.70128 (PMC12531613; doi:10.1002/ardp.70128)

**Supplemental Material: Novel Compounds**

NMR spectra: Avance III HD 400 MHz Bruker BioSpin (^1^H: 400 MHz, ^13^C: 100 MHz); 500 MHz Avance III HD 500 MHz Bruker BioSpin (^1^H: 500 MHz, ^13^C: 125 MHz); All measurements were taken in deuterated solvents. Chemical shift referencing by tetramethylsilane (TMS) as internal standard or using the solvent signal for calibration.

*tert*-Butyl 4-(3,4-dihydroisoquinolin-2(1*H*)-yl)piperidine-1-carboxylate (3a)

*tert*-Butyl 4-(6,7-dimethoxy-3,4-dihydroisoquinolin-2(1*H*)-yl)piperidine-1-carboxylate (3b)

*tert*-Butyl 4-(octahydroisoquinolin-2(1*H*)-yl)piperidine-1-carboxylate (3c)

2-(Piperidin-4-yl)-1,2,3,4-tetrahydroisoquinoline (4a)

6,7-Dimethoxy-2-(piperidin-4-yl)-1,2,3,4-tetrahydroisoquinoline (4b)

(±)-2-(Piperidin- 4-yl)decahydroisoquinoline (4c)

1-(4-(3,4-Dihydroisoquinolin-2(1*H*)-yl)piperidin-1-yl)octan-1-one (5b)

1-[4-(6,7-Dimethoxy-3,4-dihydroisoquinolin-2(1*H*)-yl)-piperidin-1-yl]butan-1-one (5d)

1-(4-(6,7-Dimethoxy-3,4-dihydroisoquinolin-2(1*H*)-yl)-piperidin-1-yl)-octan-1-one (5e)

1-[4-(6,7-Dimethoxy-3,4-dihydroisoquinolin-2(1*H*)-yl)piperidin-1-yl]dodecan-1-one (5f)

(±)-1-(4-(Octahydroisoquinolin-2(1*H*)-yl)-piperidin-1-yl)-butan-1-one (5g)

(±)-1-(4-(Octahydroisoquinolin-2(1H)-yl)piperidin-1-yl)octan-1-one (5h)

**(±)-1-[4-(Octahydroisoquinolin-2(1H)-yl)-piperidin-1-yl]-dodecan-1-one (5i)**

2-(1-Octylpiperidin-4-yl)-1,2,3,4-tetrahydroisoquinoline (6b)

2-(1-Butylpiperidin-4-yl)-6,7-dimethoxy-1,2,3,4-tetrahydroisoquinoline (6d)

6,7-Dimethoxy-2-(1-octylpiperidin-4-yl)-1,2,3,4-tetrahydroisoquinoline (6e)

2-(1-Dodecylpiperidin-4-yl)-6,7-dimethoxy-1,2,3,4-tetrahydroisoquinoline (6f)

(±)-2-(1-Butylpiperidin-4-yl)-decahydroisoquinoline (6g)

**(±)-2-(1-Octylpiperidin-4-yl)decahydroisoquinoline (6h)**

(±)-2-(1-Dodecylpiperidin-4-yl)-decahydroisoquinoline (6i)

(±)-2-[1-(3,7-Dimethyloct-6-en-1-yl)piperidin-4-yl]-6,7-dimethoxy-1,2,3,4-tetrahydroisoquinoline (6j)

(±)-2-[1-(3,7-Dimethyloct-6-en-1-yl)-piperidin-4-yl]-decahydroisoquinoline (6k)

2-{1-[(2*E*)-3,7-Dimethylocta-2,6-dien-1-yl]piperidin-4-yl}-decahydroisoquinoline (6l)

**2-(1-Benzylpiperidin-4-yl)-1,2,3,4-tetrahydroisoquinoline (7a)**

2-(1-Benzylpiperidin-4-yl)-6,7-dimethoxy-1,2,3,4-tetrahydroisoquinoline (7b)

2-(1-Benzylpiperidin-4-yl)decahydroisoquinoline (7c)

<http://www.swissadme.ch/index.php>

2-(1-Octylpiperidin-4-yl)-1,2,3,4-tetrahydroisoquinoline (6b)


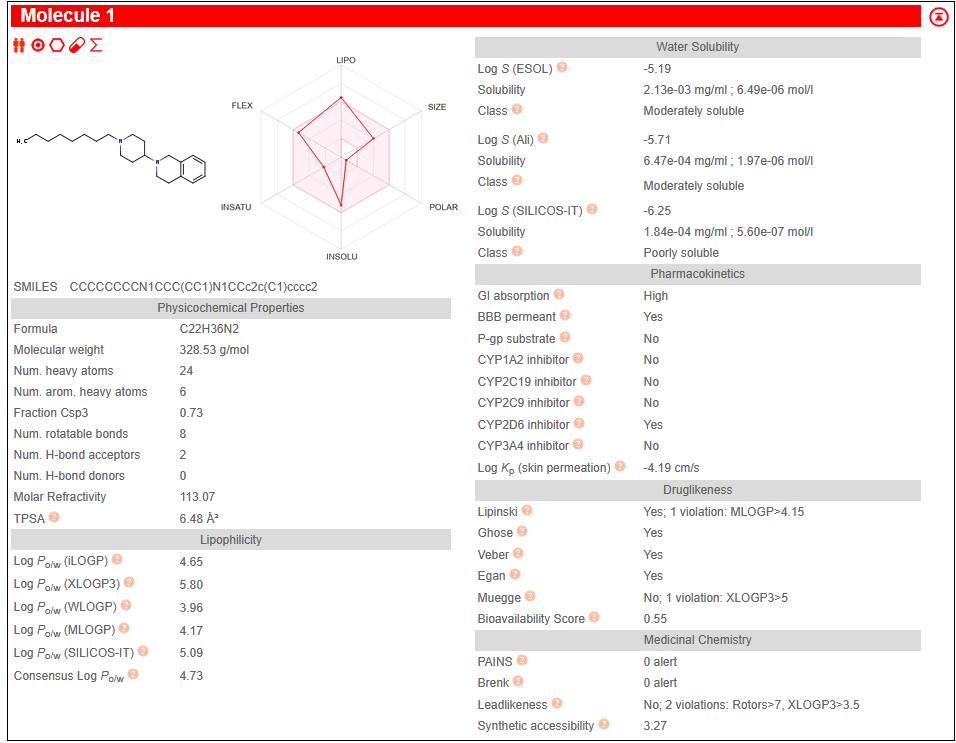


**2-(1-Butylpiperidin-4-yl)-6,7-dimethoxy-1,2,3,4-tetrahydroisoquinoline (6d)**


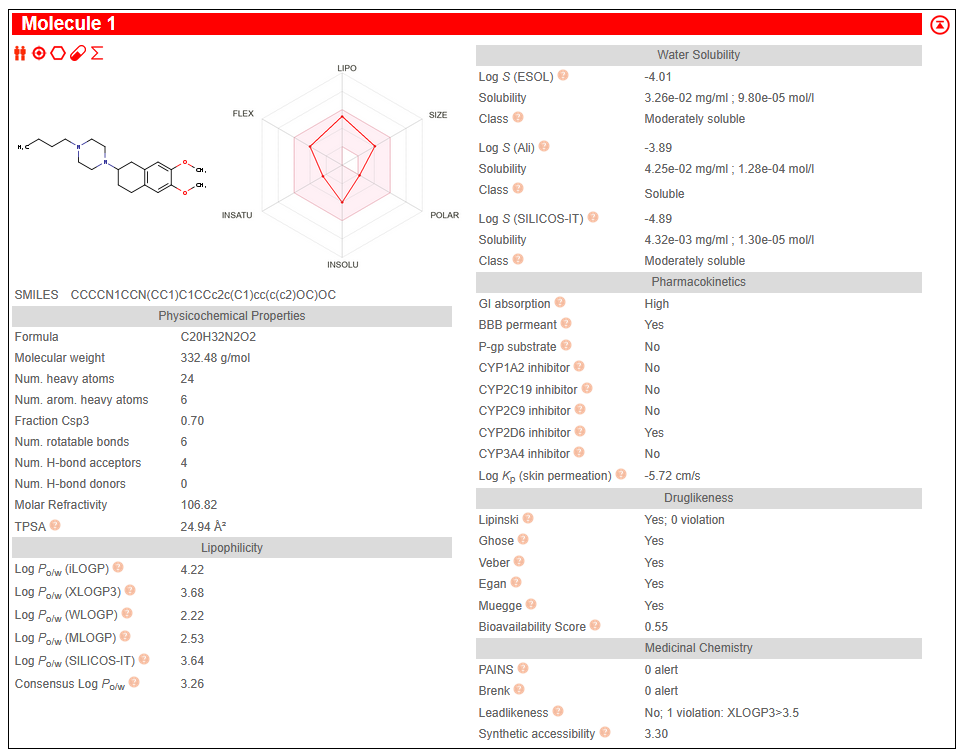


6,7-Dimethoxy-2-(1-octylpiperidin-4-yl)-1,2,3,4-tetrahydroisoquinoline (6e)


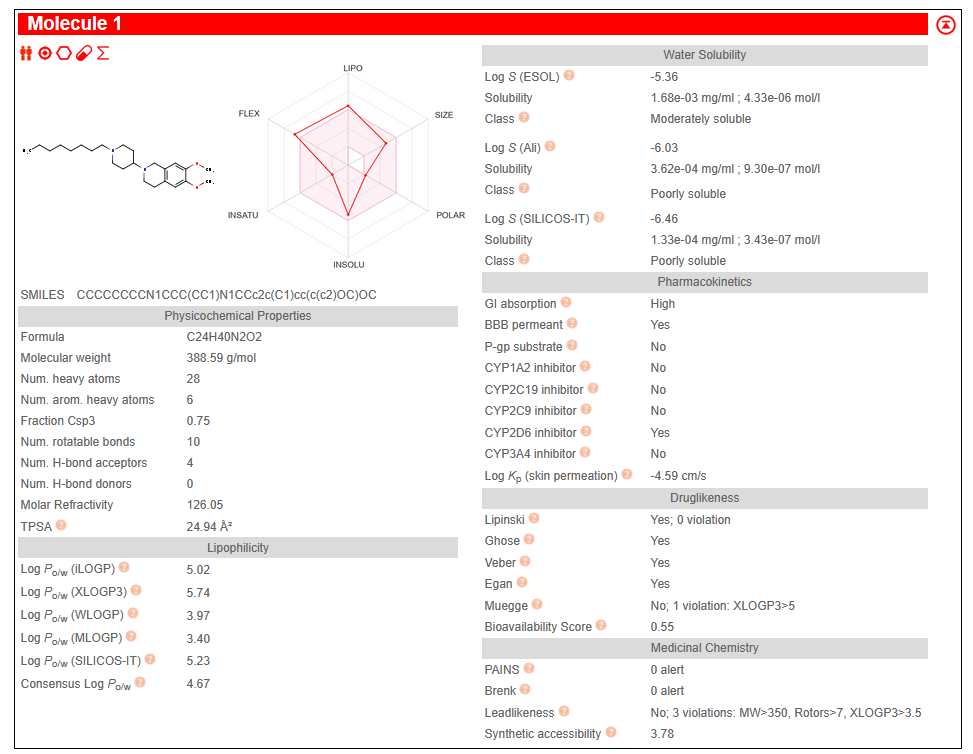


2-(1-Dodecylpiperidin-4-yl)-6,7-dimethoxy-1,2,3,4-tetrahydroisoquinoline (6f)


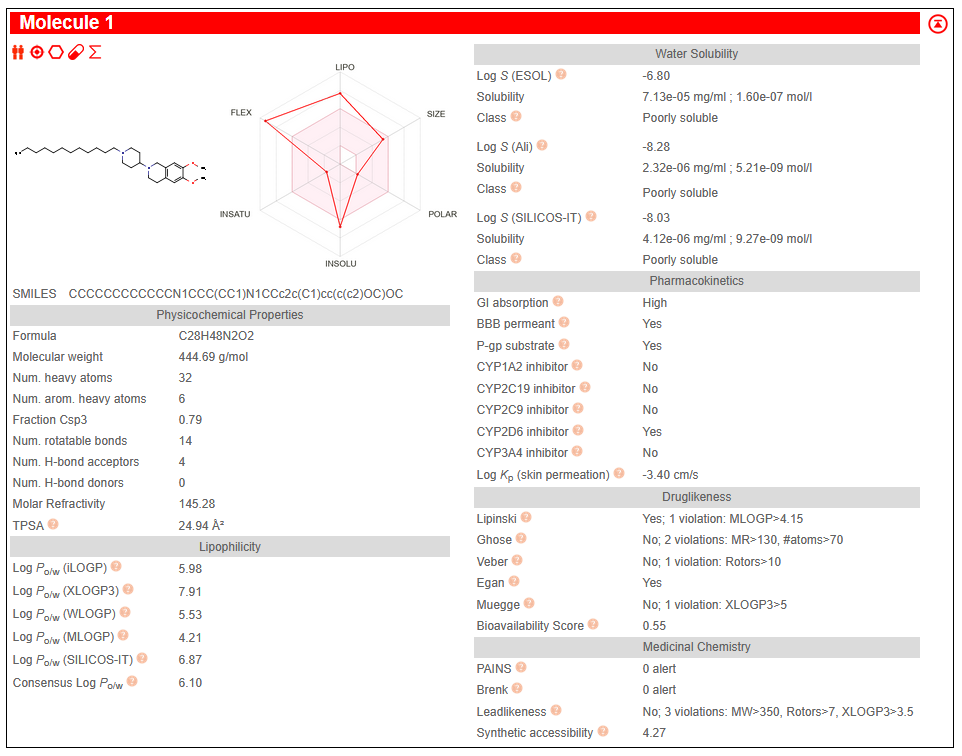


(±)-2-(1-Butylpiperidin-4-yl)-decahydroisoquinoline (6g)


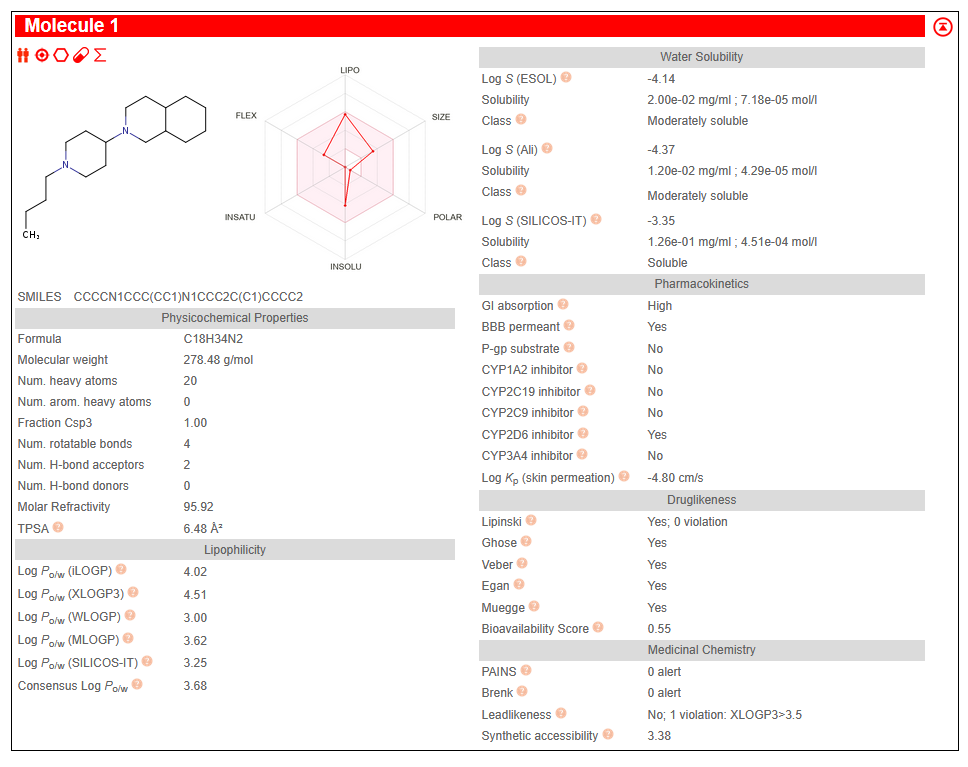


(±)-2-(1-Octylpiperidin-4-yl)decahydroisoquinoline (6h)


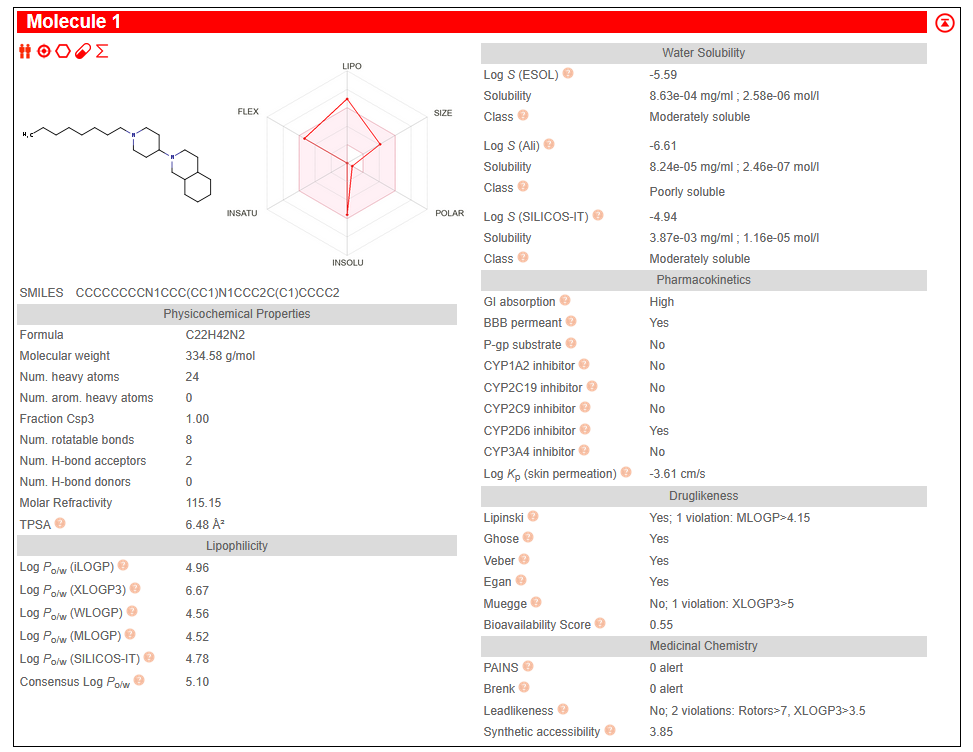


(±)-2-(1-Dodecylpiperidin-4-yl)-decahydroisoquinoline (6i)


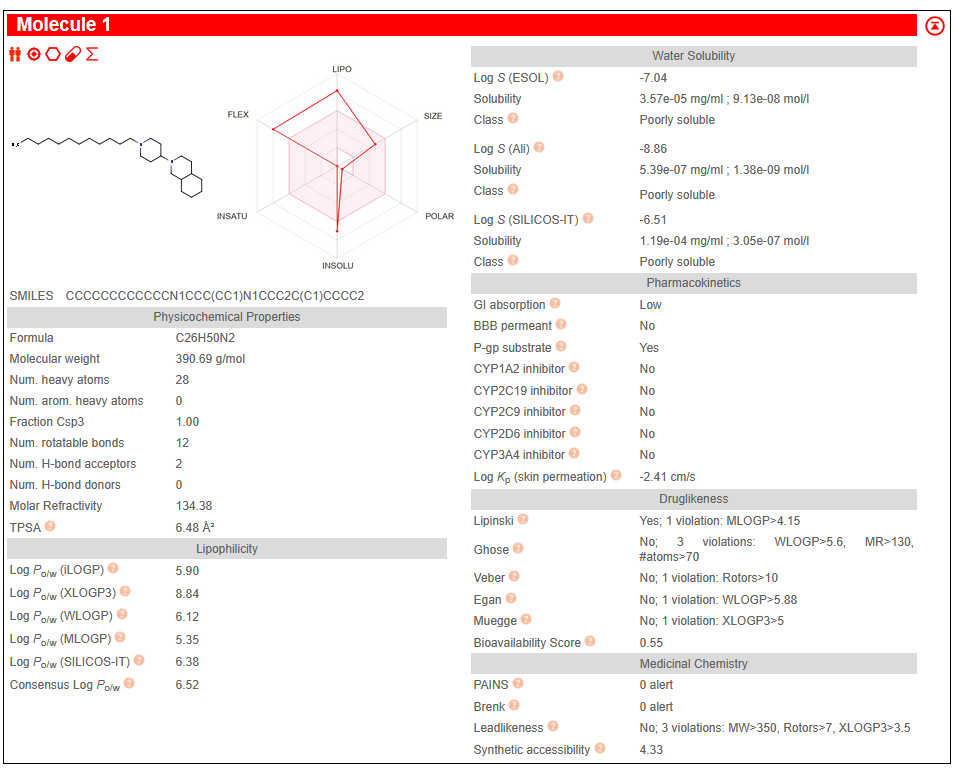


(±)-2-[1-(3,7-Dimethyloct-6-en-1-yl)piperidin-4-yl]-6,7-dimethoxy-1,2,3,4-tetrahydroisoquinoline (6j)


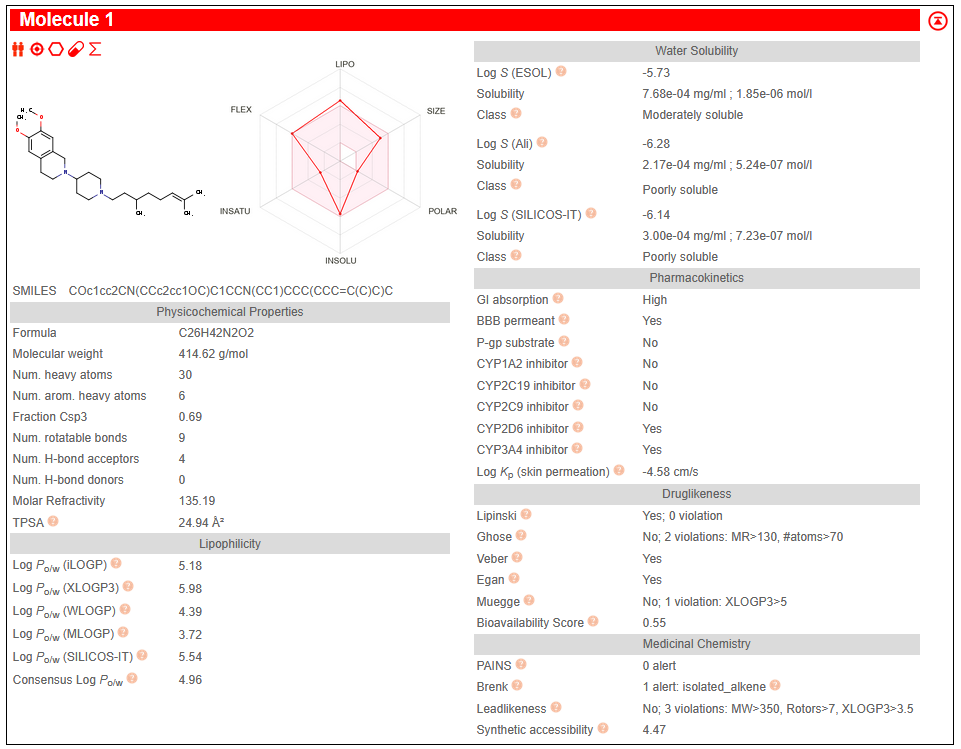


(±)-2-[1-(3,7-Dimethyloct-6-en-1-yl)-piperidin-4-yl]-decahydroisoquinoline (6k)


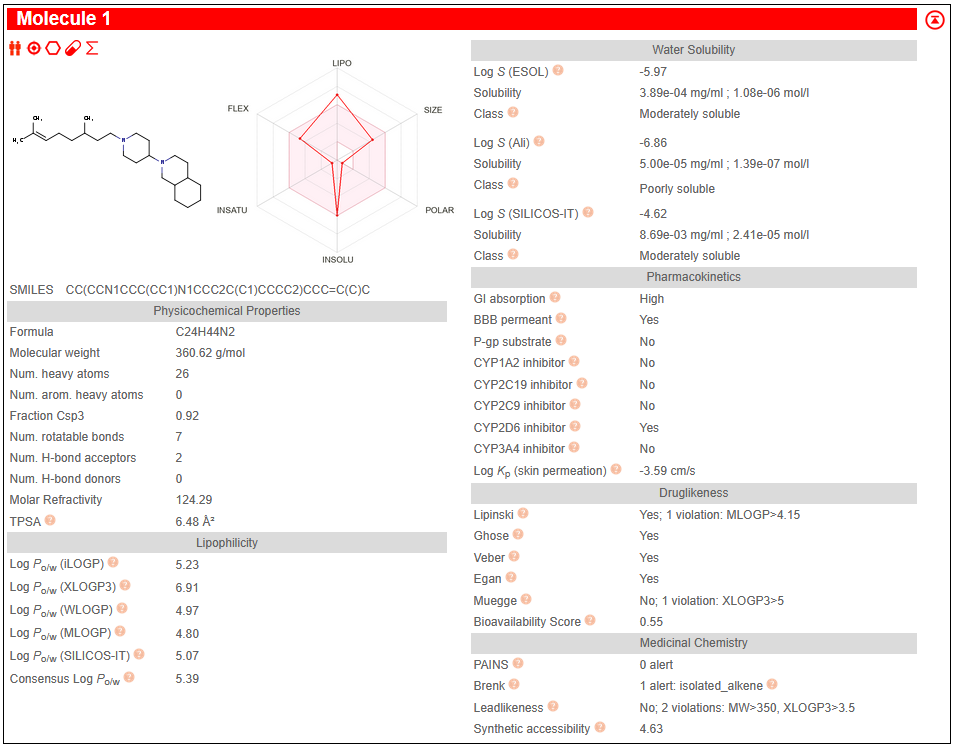


2-{1-[(2*E*)-3,7-Dimethylocta-2,6-dien-1-yl]piperidin-4-yl}-decahydroisoquinoline (6l)


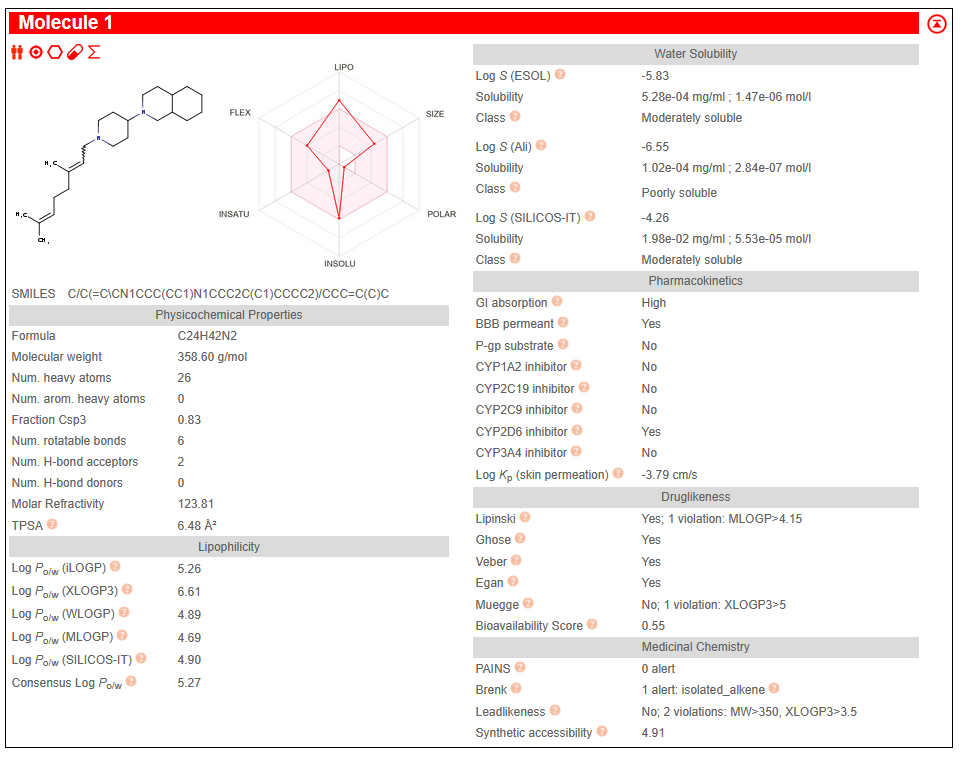


2-(1-Benzylpiperidin-4-yl)-1,2,3,4-tetrahydroisoquinoline (7a)


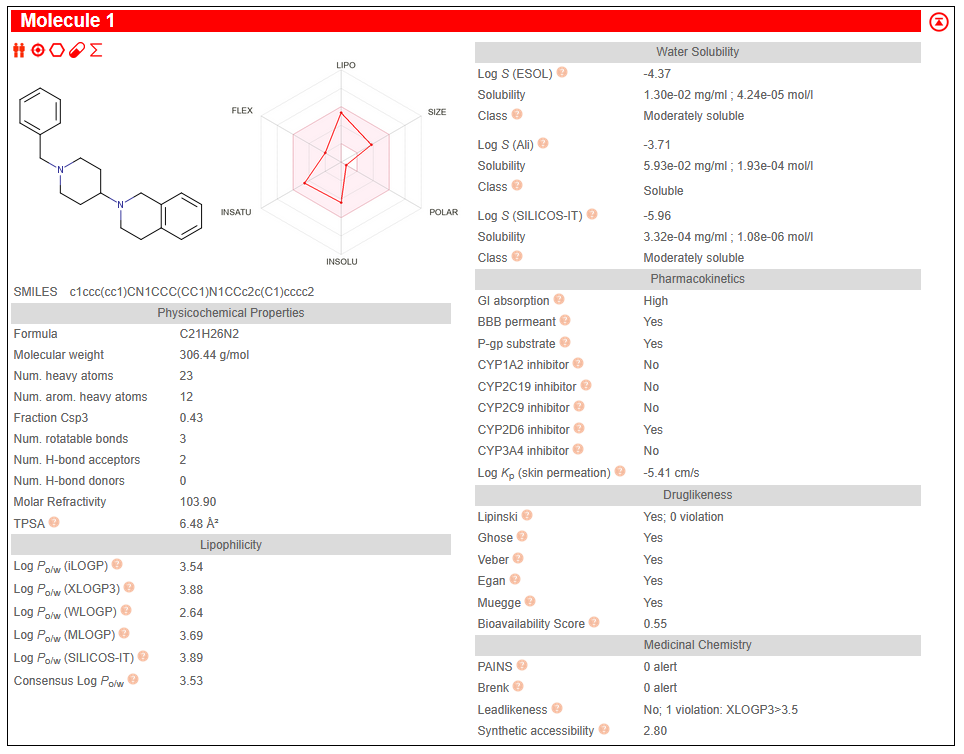


2-(1-Benzylpiperidin-4-yl)-6,7-dimethoxy-1,2,3,4-tetrahydroisoquinoline (7b)


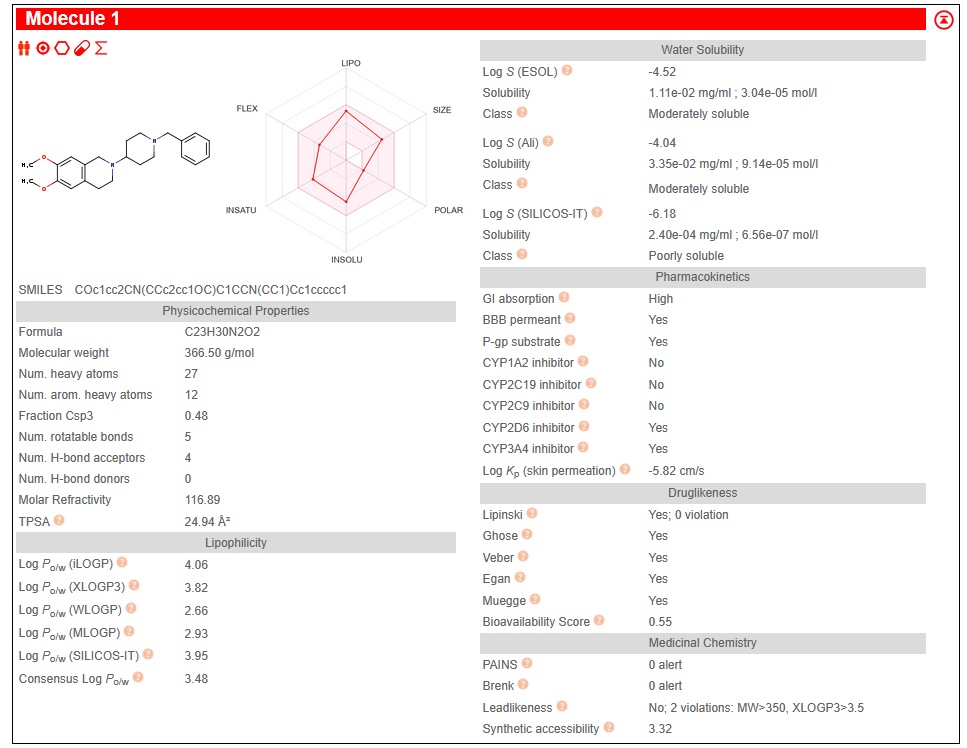


2-(1-Benzylpiperidin-4-yl)decahydroisoquinoline (7c)


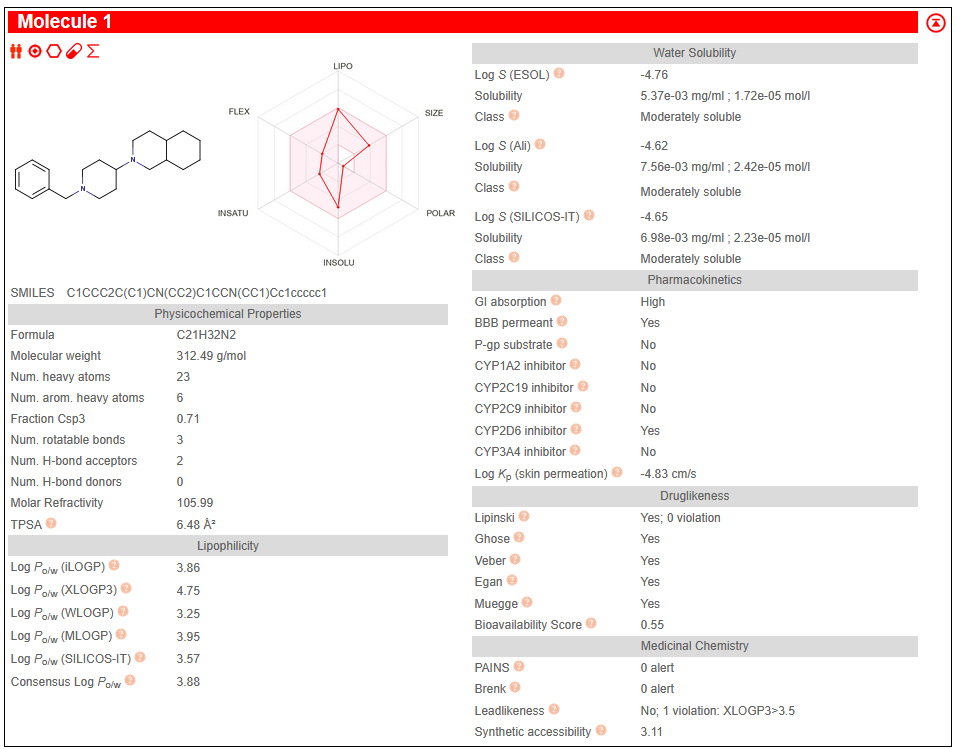

Supplement: Supplementary file 1 — ArchPharm SupplMat NMR FB. [file ARDP-358-e70128-s002.doc]
