# Supplementary material for: Synthesis and Biological Evaluation of Novel 2‐(Piperidin‐4‐yl)‐1,2,3,4‐tetrahydroisoquinoline and 2‐(Piperidin‐4‐yl)decahydroisoquinoline Antimycotics
Source: Arch Pharm (Weinheim). 2025 Oct 16;358(10):e70128. doi: 10.1002/ardp.70128 (PMC12531613; doi:10.1002/ardp.70128)
Supplement: Supplementary file 2 — Krauss et al InChI. [file ARDP-358-e70128-s001.doc]

**Supplemental Material: Novel Compounds and Biological Screening Results**

Synthesis and biological evaluation of novel 2-(piperidin-4-yl)-1,2,3,4-tetrahydroisoquinoline and 2-(piperidin-4-yl)decahydroisoquinoline antimycotics

M. Hain^1^, M. Klimt^1^, F. Bracher^1^, U. Binder^2*^, J. Krauß^1*^

1 Department of Pharmacy, Center of Drug Research, LMU Munich, Butenandtstr. 5-13, 81377 Munich, Germany.

2 Institute of Hygiene and Medical Microbiology, Department of Hygiene, Microbiology and Public Health, Medical University Innsbruck, Schöpfstr. 41, 6020 Innsbruck, Austria.

*Correspondence:

juergen.krauss@cup.uni-muenchen.de

ulrike.binder@i-med.ac.at

| **Compound No.** | **InChI** | **Biological Activity**  **MIC [µg/mL]**  ***Yarrowia lipolytica*** |
| --- | --- | --- |
| **3a** | InChI=1S/C19H28N2O2/c1-19(2,3)23-18(22)20-12-9-17(10-13-20)21-11-8-15-6-4-5-7-16(15)14-21/h4-7,17H,8-14H2,1-3H3 | >100 |
| **3b** | InChI=1S/C21H32N2O4/c1-21(2,3)27-20(24)22-10-7-17(8-11-22)23-9-6-15-12-18(25-4)19(26-5)13-16(15)14-23/h12-13,17H,6-11,14H2,1-5H3 | >100 |
| **3c** | InChI=1S/C19H34N2O2/c1-19(2,3)23-18(22)20-12-9-17(10-13-20)21-11-8-15-6-4-5-7-16(15)14-21/h15-17H,4-14H2,1-3H3 | >100 |
| **4a** | InChI=1S/C14H20N2/c1-2-4-13-11-16(10-7-12(13)3-1)14-5-8-15-9-6-14/h1-4,14-15H,5-11H2 | n.t. |
| **4b** | InChI=1S/C16H24N2O2/c1-19-15-9-12-5-8-18(14-3-6-17-7-4-14)11-13(12)10-16(15)20-2/h9-10,14,17H,3-8,11H2,1-2H3 | > 100 |
| **4c** | InChI=1S/C14H26N2/c1-2-4-13-11-16(10-7-12(13)3-1)14-5-8-15-9-6-14/h12-15H,1-11H2 | > 100 |
| **5b** | InChI=1S/C22H34N2O/c1-2-3-4-5-6-11-22(25)23-16-13-21(14-17-23)24-15-12-19-9-7-8-10-20(19)18-24/h7-10,21H,2-6,11-18H2,1H3 | > 100 |
| **5d** | InChI=1S/C20H30N2O3/c1-4-5-20(23)21-10-7-17(8-11-21)22-9-6-15-12-18(24-2)19(25-3)13-16(15)14-22/h12-13,17H,4-11,14H2,1-3H3 | > 100 |
| **5e** | InChI=1S/C24H38N2O3/c1-4-5-6-7-8-9-24(27)25-14-11-21(12-15-25)26-13-10-19-16-22(28-2)23(29-3)17-20(19)18-26/h16-17,21H,4-15,18H2,1-3H3 | > 100 |
| **5f** | InChI=1S/C28H46N2O3/c1-4-5-6-7-8-9-10-11-12-13-28(31)29-18-15-25(16-19-29)30-17-14-23-20-26(32-2)27(33-3)21-24(23)22-30/h20-21,25H,4-19,22H2,1-3H3 | > 100 |
| **5g** | InChI=1S/C18H32N2O/c1-2-5-18(21)19-12-9-17(10-13-19)20-11-8-15-6-3-4-7-16(15)14-20/h15-17H,2-14H2,1H3 | > 100 |
| **5h** | InChI=1S/C22H40N2O/c1-2-3-4-5-6-11-22(25)23-16-13-21(14-17-23)24-15-12-19-9-7-8-10-20(19)18-24/h19-21H,2-18H2,1H3 | > 100 |
| **5i** | InChI=1S/C26H48N2O/c1-2-3-4-5-6-7-8-9-10-15-26(29)27-20-17-25(18-21-27)28-19-16-23-13-11-12-14-24(23)22-28/h23-25H,2-22H2,1H3 | > 100 |
| **6b** | InChI=1S/C22H36N2/c1-2-3-4-5-6-9-15-23-16-13-22(14-17-23)24-18-12-20-10-7-8-11-21(20)19-24/h7-8,10-11,22H,2-6,9,12-19H2,1H3 | 100 |
| **6d** | InChI=1S/C20H32N2O2/c1-4-5-9-21-10-7-18(8-11-21)22-12-6-16-13-19(23-2)20(24-3)14-17(16)15-22/h13-14,18H,4-12,15H2,1-3H3 | > 100 |
| **6e** | InChI=1S/C24H40N2O2/c1-4-5-6-7-8-9-13-25-14-11-22(12-15-25)26-16-10-20-17-23(27-2)24(28-3)18-21(20)19-26/h17-18,22H,4-16,19H2,1-3H3 | 1.6 |
| **6f** | InChI=1S/C28H48N2O2/c1-4-5-6-7-8-9-10-11-12-13-17-29-18-15-26(16-19-29)30-20-14-24-21-27(31-2)28(32-3)22-25(24)23-30/h21-22,26H,4-20,23H2,1-3H3 | 3.1 |
| **6g** | InChI=1S/C18H34N2/c1-2-3-11-19-12-9-18(10-13-19)20-14-8-16-6-4-5-7-17(16)15-20/h16-18H,2-15H2,1H3 | > 100 |
| **6h** | InChI=1S/C22H42N2/c1-2-3-4-5-6-9-15-23-16-13-22(14-17-23)24-18-12-20-10-7-8-11-21(20)19-24/h20-22H,2-19H2,1H3 | 3.1 |
| **6i** | InChI=1S/C26H50N2/c1-2-3-4-5-6-7-8-9-10-13-19-27-20-17-26(18-21-27)28-22-16-24-14-11-12-15-25(24)23-28/h24-26H,2-23H2,1H3 | 1.6 |
| **6j** | InChI=1S/C26H42N2O2/c1-20(2)7-6-8-21(3)9-13-27-14-11-24(12-15-27)28-16-10-22-17-25(29-4)26(30-5)18-23(22)19-28/h7,17-18,21,24H,6,8-16,19H2,1-5H3 | 3.1 |
| **6k** | InChI=1S/C24H44N2/c1-20(2)7-6-8-21(3)11-15-25-16-13-24(14-17-25)26-18-12-22-9-4-5-10-23(22)19-26/h7,21-24H,4-6,8-19H2,1-3H3 | 6.3 |
| **6l** | InChI=1S/C24H42N2/c1-20(2)7-6-8-21(3)11-15-25-16-13-24(14-17-25)26-18-12-22-9-4-5-10-23(22)19-26/h7,11,22-24H,4-6,8-10,12-19H2,1-3H3/b21-11+ | 12.5 |
| **7a** | InChI=1S/C21H26N2/c1-2-6-18(7-3-1)16-22-13-11-21(12-14-22)23-15-10-19-8-4-5-9-20(19)17-23/h1-9,21H,10-17H2 | > 100 |
| **7b** | InChI=1S/C23H30N2O2/c1-26-22-14-19-8-13-25(17-20(19)15-23(22)27-2)21-9-11-24(12-10-21)16-18-6-4-3-5-7-18/h3-7,14-15,21H,8-13,16-17H2,1-2H3 | >100 |
| **7c** | InChI=1S/C21H32N2/c1-2-6-18(7-3-1)16-22-13-11-21(12-14-22)23-15-10-19-8-4-5-9-20(19)17-23/h1-3,6-7,19-21H,4-5,8-17H2 | > 100 |

n.t. not tested

Determination of MICs (minimal inhibitory concentration) against *Yarrowia lipolytica* was carried out according to Lit.^[13]^ Concentration of yeast cells was determined by photometer and adjusted to a turbitity of 0.5 according to McFarland Standard. MIC_100_ was determined by microdilution plate reader (Tecan) at 600 nm, after 48 h incubation at 25°C.

[13] J. Krauß, C. Müller, M. Klimt, L. J. Valero, J. F. Martínez, M. Müller, K. Bartel, U. Binder, F. Bracher, Molecules 2021, 26, 7208. https://doi.org/10.3390/molecules26237208
